# A novel mechanism for morphological change during bacterial sporulation based on programmed peptidoglycan degradation

**DOI:** 10.7554/eLife.108250

**Published:** 2026-11-05

**Authors:** Carlos A Ramirez Carbo, Oihane Irazoki, Srutha Venkatesan, Lauren JS Chen, Haylie A Morales, Assariel J Garcia Avila, Hoi-Ling Cheung, Felipe Cava, Beiyan Nan

**Affiliations:** 1 Department of Biology, Texas A&M University College Station United States; 2 The Genetics and Genomics Interdisciplinary Program, Texas A&M University College Station United States; 3 Department of Molecular Biology and Laboratory for Molecular Infection Medicine Sweden, Umeå Centre for Microbial Research, SciLifeLab, Umeå University Umeå Sweden; University of the Witwatersrand South Africa; University of the Witwatersrand South Africa

**Keywords:** lytic transglycosylases, Gram-negative sporulation, peptidoglycan, cell wall dynamics, bacterial morphology, Other

## Abstract

Many bacteria form spores to endure unfavorable conditions. While *Firmicutes* generate endospores through cell division, sporulation in non-Firmicutes remains less understood. The Gram-negative bacterium *Myxococcus xanthus* undergoes sporulation through two distinct mechanisms: rapid sporulation triggered by chemical induction and slow sporulation driven by starvation, both occurring independently of cell division. Instead, these processes depend on the complete degradation of the peptidoglycan (PG) cell wall by two lytic transglycosylases (LTGs), LtgA and LtgB. Remarkably, LtgB programs the pace of PG degradation by LtgA during rapid sporulation, ensuring a controlled process that prevents abrupt PG breakdown and the formation of non-resistant pseudospores. In addition to regulation between LTGs, PG degradation is also influenced by its synthesis; cells exhibiting increased muropeptide production often circumvent sporulation. These findings not only reveal novel mechanisms of bacterial sporulation but also shed light on the regulatory network governing PG dynamics.

## Introduction

Spores are metabolically dormant cells that can survive unfavorable conditions, including extremes of temperature, desiccation, and ionizing radiation (Huang and Hull, 2017; Hutchison et al., 2014). Sporulation, the process of spore development, is a strategy utilized by a wide variety of organisms, from bacteria and protozoa to fungi and plants. In addition to protective spore coats, spore resilience relies on cytosol dehydration and DNA compaction—key processes that decrease cell volume and alter cell shape (Higgins and Dworkin, 2012). Thus, morphological differentiation is a hallmark of sporulation. In bacterial spore formers, as peptidoglycan (PG) cell walls largely determine cell morphology, their sporulation requires profound PG remodeling.

PG is a rigid, mesh-like macromolecule that is composed of glycan strands of repeating units of N-acetyl glucosamine (GluNAc)-N-acetyl muramic acid (MurNAc) crosslinked by peptides (Egan et al., 2020). PG encloses the entire cytoplasmic membrane, and its rigidity provides mechanical support against osmotic stress. For this reason, PG is an essential structure for most bacteria and a major target for antibacterial treatments. Sporulation provides invaluable opportunities for understanding the dynamics of PG, which is controlled by multiple synthases and hydrolases. PG synthases include glycosyltransferases (GTases) that polymerize glycan strands and transpeptidases (TPases) that form peptide crosslinks (Egan et al., 2020). PG hydrolases, also known as autolysins, include glycosidases that break the glycan strands, and amidases and endo/carboxypeptidases that cleave the peptide crosslinks (Egan et al., 2020; Rohs and Bernhardt, 2021; van Heijenoort, 2011).

*Firmicutes*, including Gram-positive bacteria like *Bacilli* and *Clostridia*, produce endospores. Their morphological transition from rod-shaped vegetative cells to ovoid spores occurs through asymmetric cell division, resulting in the formation of a smaller forespore and a larger mother cell. Eventually, the forespore becomes an oval endospore after being engulfed by the mother cell (Higgins and Dworkin, 2012; McKenney and Eichenberger, 2012). A mature endospore contains two PG layers: the germ cell wall, derived from the forespore’s original PG, and a thickened PG cortex deposited by the mother cell (Popham and Bernhards, 2015). While a few Gram-negative spore formers also belong to the *Firmicutes* phylum, they share conserved sporulation genes with *Bacilli* and *Clostridia*, which suggests similar sporulation mechanisms (Tocheva et al., 2011; Yutin and Galperin, 2013).

Sporulation by non-firmicutes bacteria has been largely overlooked. Myxobacteria, a group of Gram-negative, non-firmicutes spore formers, were first assigned to the phylum δ-proteobacteria, but recently reclassified into the newly established phylum *Myxococcota* (Waite et al., 2020). *Myxococcus xanthus* is a model organism of myxobacteria. Rod-shaped *M. xanthus* cells can undergo sporulation via two distinct pathways, both resulting in spherical spores (Kroos et al., 2025). First, in response to certain chemical signals, such as glycerol and dimethyl sulfoxide (DMSO), individual *M. xanthus* cells can rapidly transition into isolated spherical spores in aqueous environments within hours (Dworkin and Gibson, 1964). Second, millions of cells can aggregate on solid starvation media and develop into spore-filled fruiting bodies, a process that takes a few days to complete (O’Connor and Zusman, 1988). Both sporulation pathways are programmed, tightly controlled processes that involve over 1000 genes (Müller et al., 2010; Muñoz-Dorado et al., 2019). In contrast to endospore formation, cell division is not involved in either the *M. xanthus* sporulation mechanisms, and *M. xanthus* lacks the homologs of the sporulation genes in *Firmicutes* (Aramayo and Nan, 2022). Thus, sporulating *M. xanthus* must accomplish the rod-to-sphere transition through yet-to-be-discovered PG remodeling mechanisms, which provide invaluable opportunities to investigate PG dynamics (Zhang et al., 2021).

Distinct from *Bacilli* and *Clostridia*, *M. xanthus* spores lack cortex PG (Bui et al., 2009; Zhang et al., 2020; Voelz and Dworkin, 1962). Rather, their resistance is derived from polysaccharide coats (Kottel et al., 1975; Pérez-Burgos et al., 2020; Saïdi et al., 2021; Müller et al., 2012). Sporulating cells that fail to deposit coat polysaccharides on their surfaces produce spherical pseudospores that lack resistance (Wartel et al., 2013; Zhang et al., 2020; Holkenbrink et al., 2014). In a pioneering study, Bui et al. did not detect muropeptides in glycerol-induced spores, indicating that *M. xanthus* degrades its PG during rapid sporulation (Bui et al., 2009). However, it remains unclear whether starvation-induced spores within fruiting bodies still retain PG and how *M. xanthus* orchestrates programmed morphological changes.

In this report, we used ultra-performance liquid chromatography (UPLC) to demonstrate that *M. xanthus* degrades PG in both sporulation pathways. Through mutagenesis studies, we identified LtgA and LtgB, two lytic transglycosylases (LTGs) essential for these sporulation pathways. Remarkably, LtgB regulates the pace of PG degradation by LtgA during rapid sporulation, preventing the formation of non-resistant pseudospores due to abrupt PG breakdown. This research not only uncovers novel mechanisms of sporulation in non-firmicutes but also highlights the crucial role of cross-regulation between PG hydrolases in maintaining cell integrity.

## Results

### PG is degraded in both spore types

**Figure 1.**
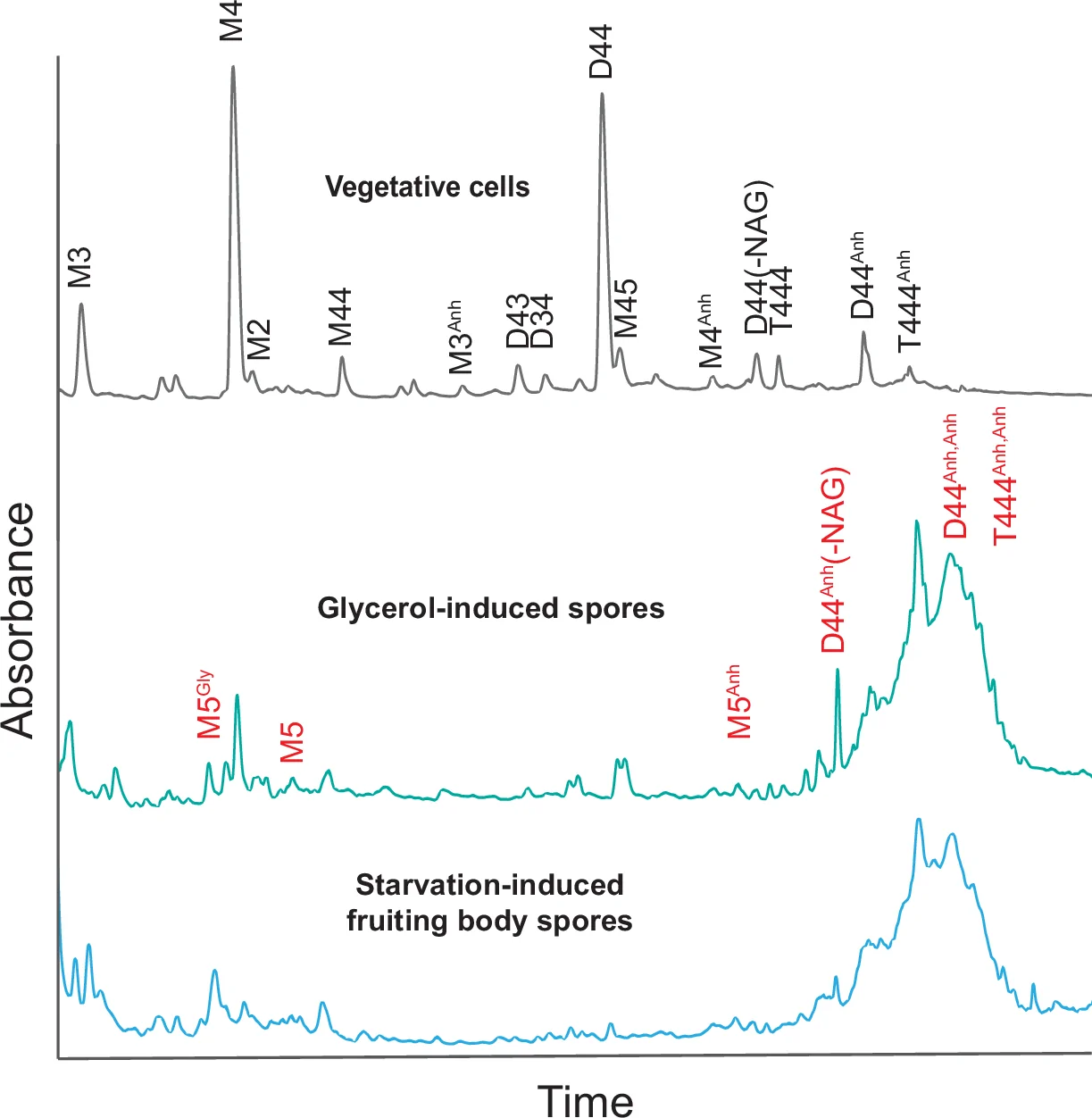
Mature *M. xanthus* spores induced by either glycerol or starvation do not contain significant muropeptides. UPLC muropeptide profiles of vegetative cells, glycerol- and starvation-induced spores indicate that the major muropeptides species, M4 and D44, in vegetative cells are diminished in both spore types, while spores are enriched in anhydro-muropeptides (Anh). Only muropeptides that could be confidently identified by MS/MS analysis are labeled. The characteristic peaks are labeled as follows: M, monomeric muropeptide (uncrosslinked); D, dimeric muropeptide (crosslink connecting two muropeptides), T, trimeric muropeptide (crosslink connecting three muropeptides). Numbers refer to the status of the peptide side chain (3, tripeptide; 4, tetrapeptide). Red characters mark the muropeptides only detected in spores.

To investigate if starvation-induced spores in *M. xanthus* fruiting bodies retain PG, we broke the fruiting bodies after 120 h of starvation and purified sacculi from spores. For comparison, we also purified the sacculi from vegetative cells and glycerol-induced spores. The purification procedure yields only sedimentable PG, as all soluble fragments are removed during the washing steps. Consequently, any PG fragments solubilized by LTG activity during sporulation would be lost at this stage, and the muropeptides we detect derive from the more highly crosslinked material that survives the procedure. The resulting sacculi were then digested with muramidase, and the solubilized muropeptides were analyzed by UPLC (see ‘Materials and methods’). The two spore types showed similar profiles of a discernible presence of muropeptides that resembled those found in vegetative cells, albeit in significantly reduced quantities (Figure 1). Importantly, the most abundant muropeptides identified in the vegetative cells, MurNAc-tetrapeptide monomer (M4) and MurNAc-tetrapeptide dimer (D44), were nearly absent in both spore types (Figure 1). Such residual muropeptides in both spore types are inadequate for forming continuous PG layers (Bui et al., 2009). This observation indicates that spore development in both pathways involves the breakdown of the vegetative PG cell wall.

Despite the overall decline in identified muropeptides, anhydro-muropeptides—a minor component of vegetative PG—were enriched in both spore types (Figure 1). Especially, anhydro-MurNAc-tetrapeptide trimer (T444^Anh^) that was only detected in trace amounts in vegetative cells, became a prominent muropeptide in both spore types (Figure 1). Moreover, new anhydro-muropeptides, including anhydro, anhydro-MurNAc-tetrapeptide dimer (D44^Anh,Anh^), anhydro, anhydro-MurNAc-tetrapeptide trimer (T444^Anh,Anh^), and an anhydrodimer without a NAG (D44^Anh^(-NAG)), were only present in spores (Figure 1). The identified muropeptides, however, represent only a part of the material released by muramidase from spores: a substantial part, as a late-eluting portion of the chromatogram could not be assigned, and its composition remains unknown. Because anhydro-muropeptides are the signature products of LTGs (Dik et al., 2017; Williams et al., 2018), their abundance in spores indicates that certain LTGs may play important roles in *M. xanthus* sporulation.

### *M. xanthus* sporulation requires two LTGs

**Figure 2.**
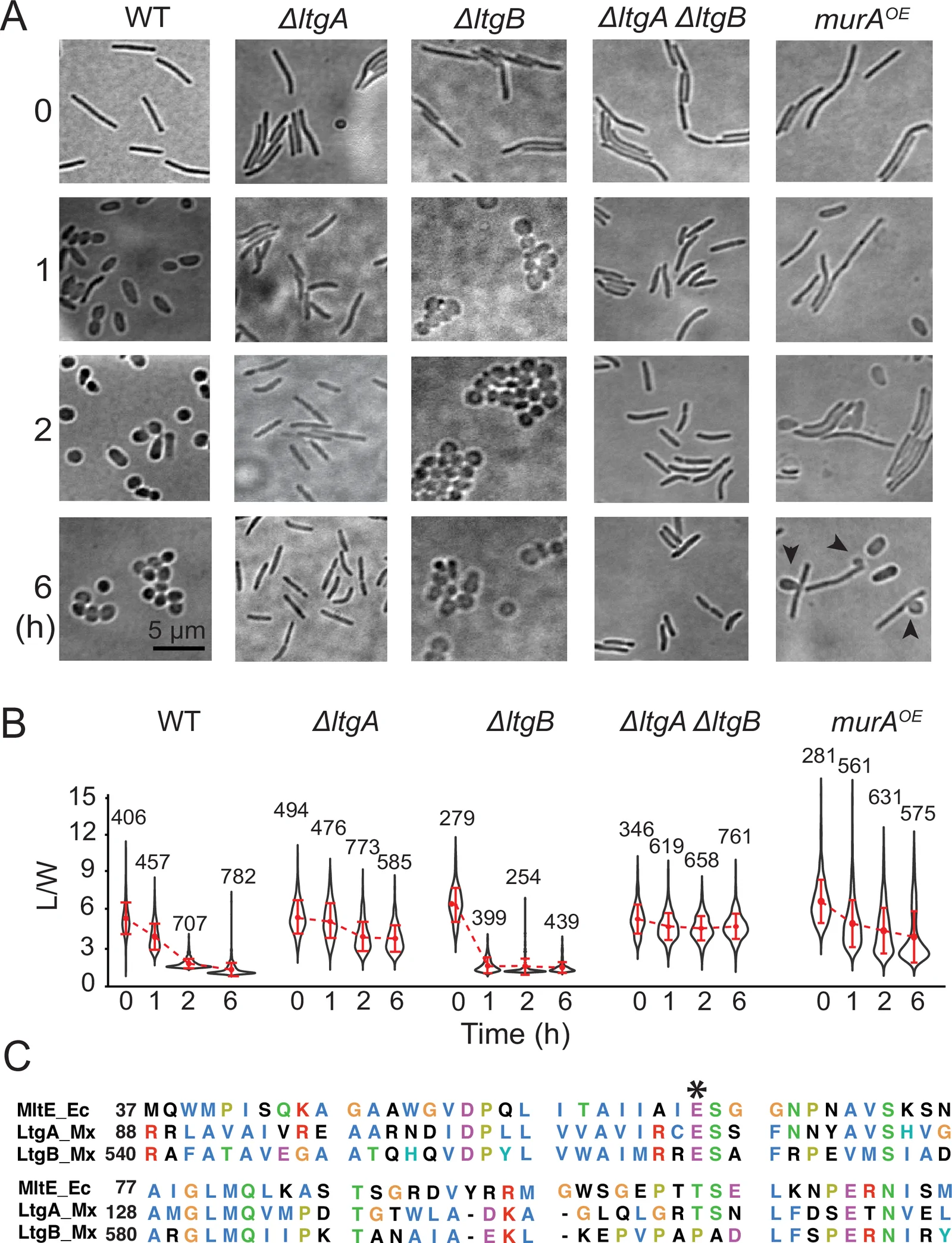
LtgA and LtgB are required for glycerol-induced sporulation, whereas MurA overexpression inhibits sporulation. (**A**) Brightfield images of cells at different time points after glycerol induction. Black arrows point to lysing cells. (**B**) Quantitative analysis of glycerol-induced sporulation using the length/width ratio (L/W) of cells. Whiskers indicate the 25th–75th percentiles and red dots the median. The total number of cells analyzed is shown on top of each plot. (**C**) LtgA and LtgB are homologous to *E. coli* MltE. The asterisk marks the conserved active site. Figure 2—source data 1.Cell morphology measurements for Figure 2B.

The genome of *M. xanthus* encodes 14 putative LTGs (Aramayo and Nan, 2022; Ramirez Carbo et al., 2024). We imaged the cells of the 14 knockout mutants (Ramirez Carbo et al., 2024) after 6 h of glycerol induction and identified two mutants that displayed abnormality in sporulation (Figure 2, Figure 2—figure supplement 1). We then imaged the wild-type and mutant cells at different time points after glycerol induction and used their length/width (L/W) ratios to monitor potential defects in the sporulation process. Wild-type cells initiated sporulation within 1 h of glycerol induction, which was evidenced by the increase in cell width and decrease in cell length (Figure 2A). Consistent with the change in cell morphology, their L/W ratios decreased continuously and stabilized after 2 h of induction, when sporulation completed. In contrast, the cells that carry the deletion of K1515_20820 (MXAN_RS16290) (Aramayo and Nan, 2022) were able to shorten cell length slightly but retained rod shape after prolonged induction (Figure 2B, Figure 2—source data 1).

**Figure 2—figure supplement 1.**
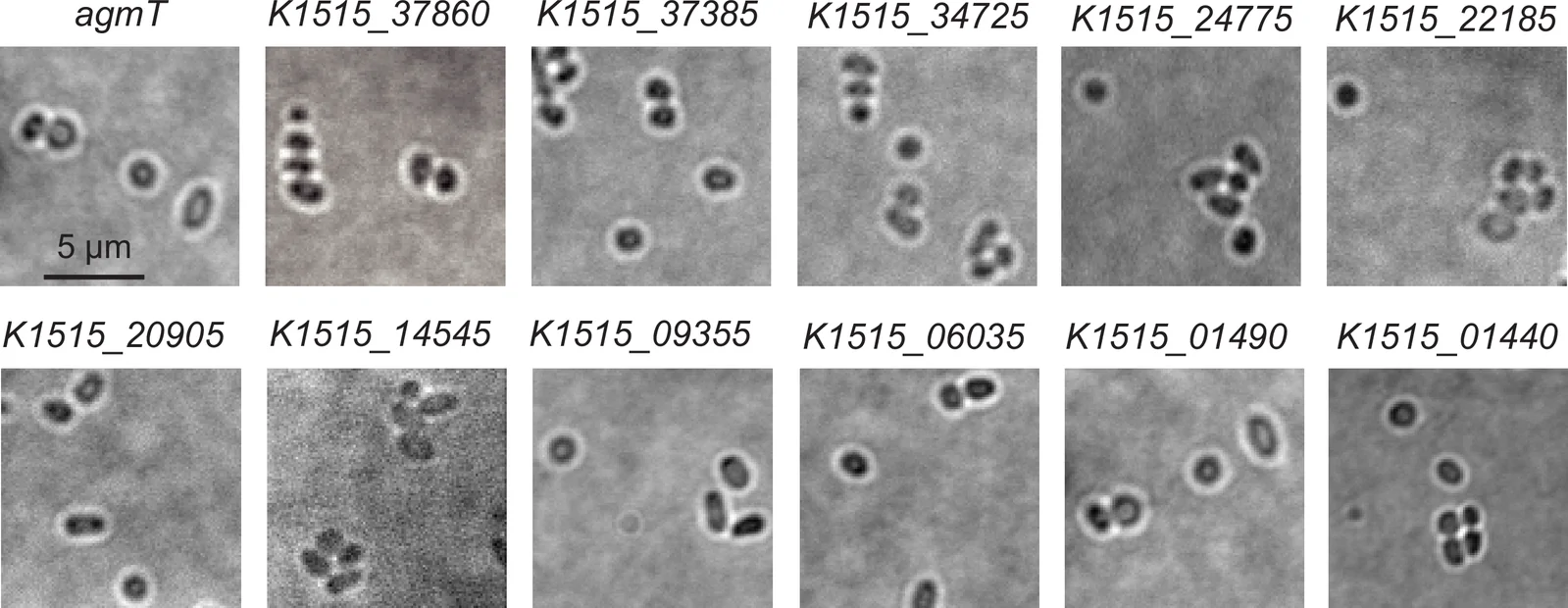
Besides LtgA and LtgB, other putative LTGs do not affect either sporulation pathway significantly. Brightfield images of cells were obtained after 4 h of glycerol induction.

**Figure 2—figure supplement 2.**
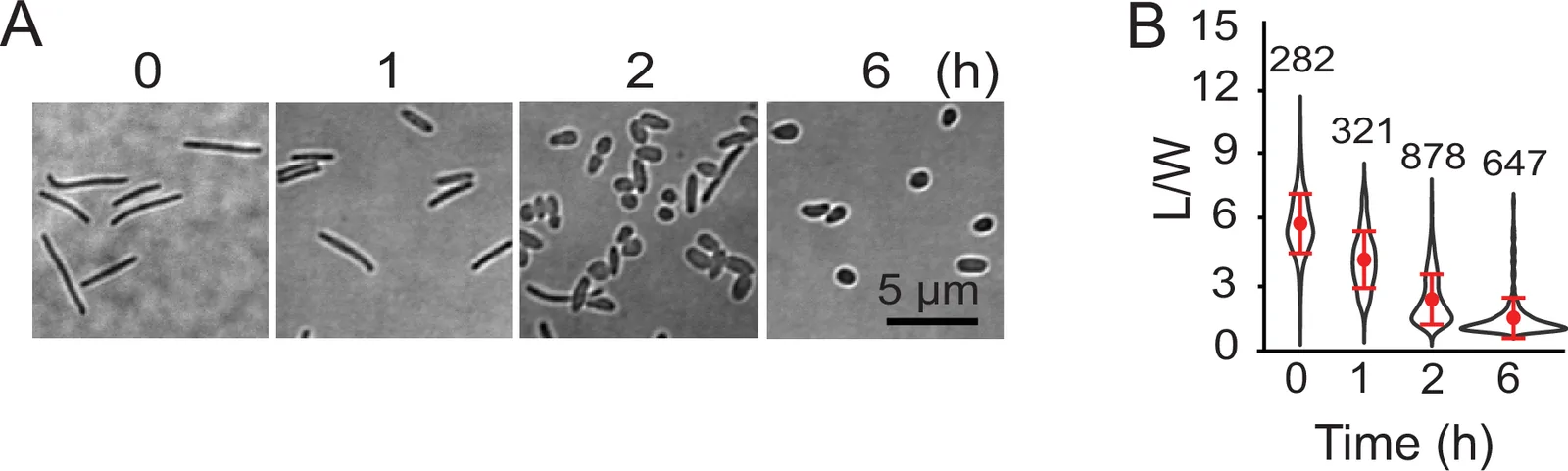
In the absence of vanillate, *P_van_*-driven MurA expression does not affect glycerol-induced sporulation. (**A**) Brightfield images of cells at different time points after glycerol induction. (**B**) Quantitative analysis of glycerol-induced sporulation using the length/width ratio (L/W) of cells. Whiskers indicate the 25th–75th percentiles and red dots the median. The total number of cells analyzed is shown on top of each plot. Figure 2—figure supplement 2—source data 1.Original cell morphology measurements for Figure 2—figure supplement 2B.

Surprisingly, another mutant that carries the deletion of K1515_17460 (MXAN_RS19615) started to lose rod shape immediately after glycerol induction and became spherical within 1 h (Figure 2A and B, Figure 2—source data 1). However, different from the wild-type spores that appeared dark and heterogeneous under differential interference contrast (DIC) microscopy, the spheres formed by this mutant appeared bright and homogenous, similar to the pseudospores from the *ΔaglQS* mutant that lack the motor for depositing spore coat polysaccharides onto cell surfaces (Zhang et al., 2020; Figure 2A). To test whether these spheres are real spores, we subjected them to sonication and quantified their survival rate using a Helber bacterial counting chamber. After sonication, while 91 ± 6% (calculated from three independent experiments, n>1000) of the wild-type spores appeared intact, only 3 ± 1% of the spheres produced by the K1515_17460 deletion mutant remained. Thus, this mutant indeed formed pseudospores that lacked resistance against sonication. As the products of K1515_20820 and K1515_17460 show homology to MltE, an LTG encoded by *Escherichia coli emtA* (49.0% and 38.4% sequence identity, respectively) (Figure 2C), we named them *ltgA* and *ltgB*, respectively. Both LtgA and LtgB are required for forming resistant spores via the rapid sporulation pathway, albeit playing different roles in the rod-to-sphere transition.

**Figure 3.**
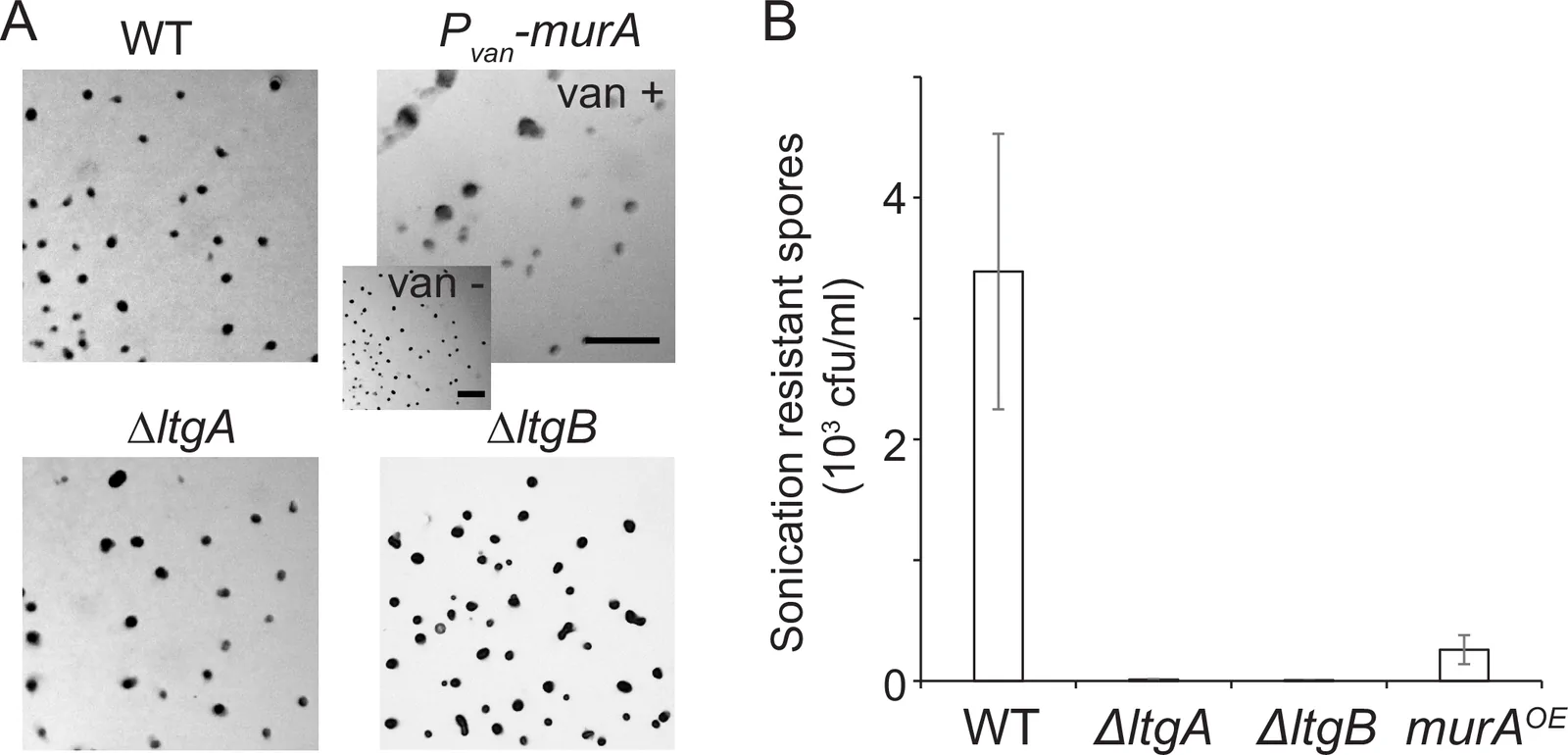
LtgA, LtgB, and MurA regulate starvation-induced sporulation. (**A**) The absence of LtgA and LtgB, and the overexpression of MurA do not affect the formation of fruiting body-like aggregates on starvation agar. The inset shows that the *P_van_*-driven MurA expression does not affect starvation-induced fruiting body formation in the absence of vanillate,. The image was taken after 96 h of incubation on a CF agar. (**B**) Both LtgA and LtgB are essential for forming starvation-induced spores, while MurA overexpression negatively regulates this process. Sonication-resistant spores were counted as colony formation units (cfu) after 96 h of incubation on CYE agar. Data are shown as mean ± standard deviation (n=3). Scale bars, 1 mm. Figure 3—source data 1.Spore counts for Figure 3B.

To determine if these LTGs are also required for starvation-induced sporulation, we grew them in rich liquid media and spotted cells on solid starvation (CF) media. After 96 h of incubation, both mutants produced dark fruiting bodies on the agar surface that were comparable to those of the wild-type (Figure 3A). To test if these mutants form starvation-induced spores, we incubated cells in static liquid CF medium using polystyrene Petri dishes for 96 h, scraped the cells attached to the dishes, suspended them in 1 ml water, subjected them to sonication, then plated them on solid rich media. After 96 h of incubation, while the wild-type fruiting bodies produced colonies of 3389±1141 cfu/ml (n=3), the *ΔltgA* and *ΔltgB* cells failed to form colonies after five days of incubation (Figure 3B, Figure 3—source data 1), indicating that both LtgA and LtgB are essential for forming starvation-induced spores.

### LtgA and LtgB play distinct roles in different sporulation pathways

**Figure 4.**
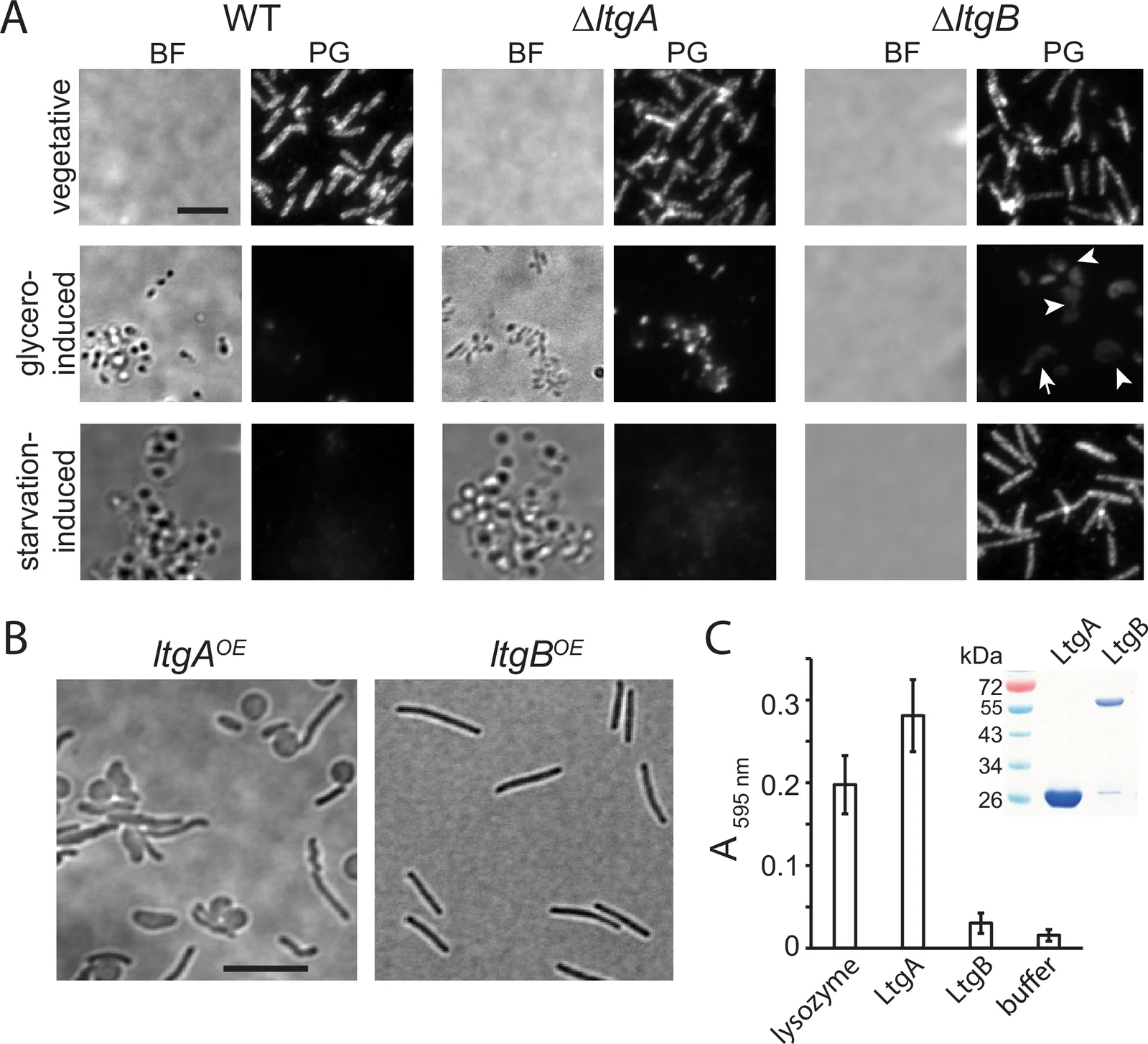
LtgA and LtgB play distinct roles in the two sporulation pathways. (**A**) While LtgA is required for PG degradation during glycerol-induced sporulation, LtgB is the major LTG for forming starvation-induced spores. PG was detected using an anti-PG serum and a fluorescence-conjugated secondary antibody. PG sacculi were purified from cells after 6 h and 120 h of glycerol-induced and starvation-induced sporulation, respectively. Flattened sacculi are not visible under brightfield microscopy. White arrows point to the sacculi in irregular shapes. BF, brightfield. (**B**) The overexpression of LtgA, but not LtgB, collapses rod shape in vegetative cells. (**C**) Purified periplasmic domain of LtgA and LtgB solubilize dye-labeled PG sacculi at different rates. Lysozyme and buffer serve as the positive and negative controls, respectively. Absorption at 595 nm was measured after 18 h incubation at 25 °C. Data are presented as mean values ± SD from three technical replicates. The inset shows purified LtgA and LtgB in a Coomassie-stained gel. Scale bars, 5 μm. Figure 4—source data 1.Unlabeled SDS-PAGE image of purified periplasmic domain of LtgA and LtgB for Figure 4C. Figure 4—source data 2.Absorption at 595 nm in the Remazol brilliant (RBB) assay for Figure 4C.

Do LtgA and LtgB degrade PG during sporulation? To answer this question, we purified cell sacculi and used immunofluorescence and an anti-PG serum (de Pedro et al., 1997) to visualize the remaining PG. The sacculi of vegetative cells from the wild-type, *∆ltgA*, and *∆ltgB* strains were not visible under DIC microscopy, likely due to their flattened structures minimizing light interference. However, PG from all three strains was readily detected in the fluorescence channel (Figure 4A). After 6 h of glycerol induction, the sacculi of both the wild-type and *∆ltgA* cells remained visible under DIC microscopy (Figure 4A), likely due to the deposition of spore coat polysaccharides that sustained unflattened cell structures (Wartel et al., 2013; Holkenbrink et al., 2014). After the sacculus purification process, only background PG signals were detected in glycerol-induced wild-type spores (Figure 4A). In contrast, the *∆ltgA* cells retained PG in substantial quantities (Figure 4A). Sacculi from the *∆ltgB* pseudospores still contained PG but showed lower fluorescence intensity (Figure 4A). While the remaining PG sacculi of these pseudospores were largely spherical, they lost integrity during purification, with many sacculi displaying irregular shapes in the fluorescence channel (Figure 4A). Thus, compared to LtgA, the enzymatic activity of LtgB plays a more limited role in PG degradation during chemical-induced sporulation. Collectively, LtgB appears to be a pace-keeper that prevents abrupt PG degradation. Different from the induced wild-type and *∆ltgA* cells, sacculi from the *∆ltgB* pseudospores were undetectable under DIC microscopy (Figure 4A), reflecting their flattened shapes. This phenotype suggests that further acceleration of the morphological transitions associated with rapid sporulation may impede the formation of a rigid polysaccharide coat.

**Figure 4—figure supplement 1.**
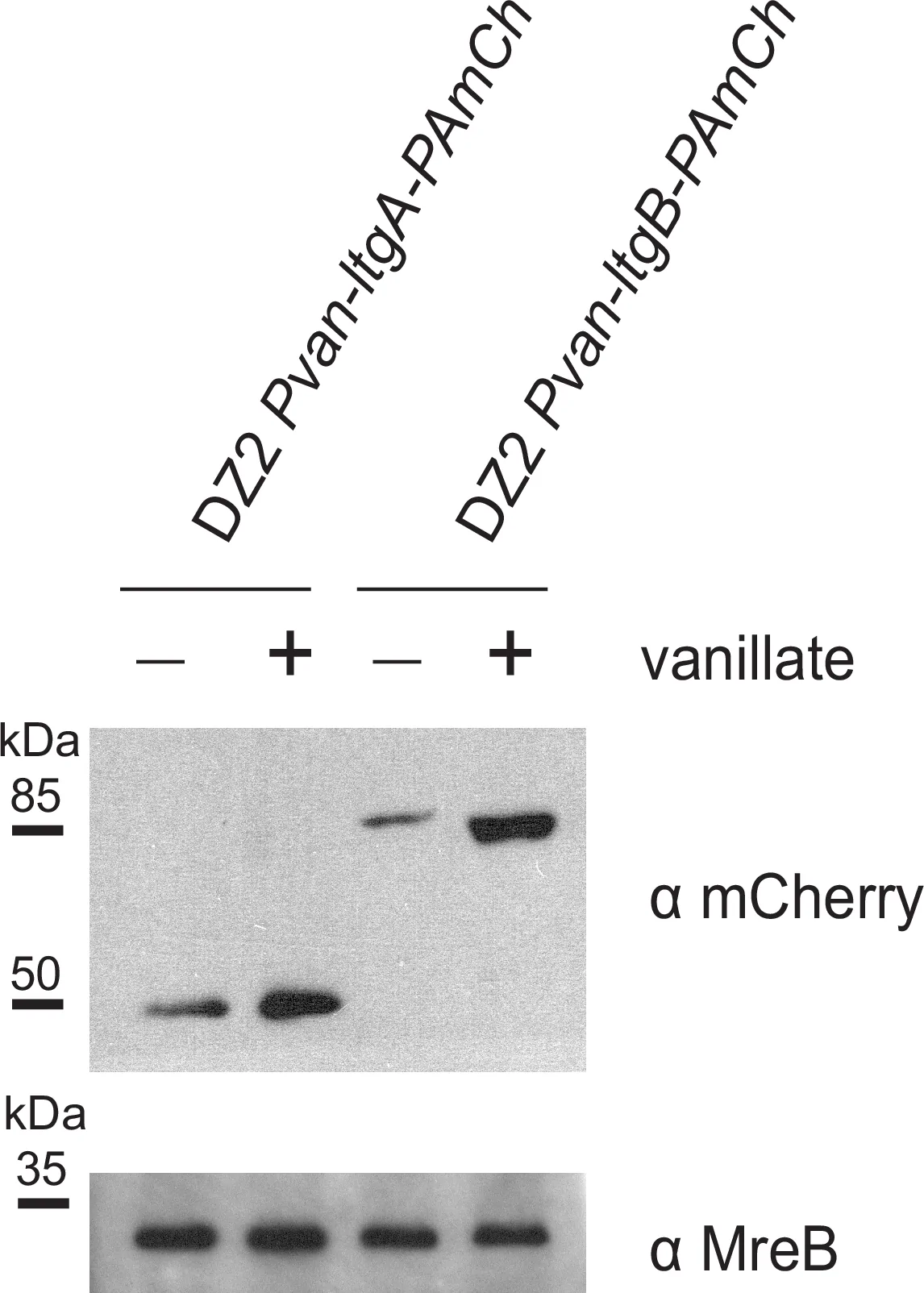
Vanillate-induced expression of LtgA-PAmCherry and LtgB-PAmCherry. Protein expression was visualized through immunoblotting using mCherry antibodies (α mCherry). The MreB protein was used as the loading control, detected by the MreB antibodies (α MreB). ‘—’, no vanillate; ‘+’, 200 μM sodium vanillate. Figure 4—figure supplement 1—source data 1.Unlabeled western blot images. Figure 4—figure supplement 1—source data 2.Labeled western blot images. Figure 4—figure supplement 1—source data 3.Original western images for Figure 4—figure supplement 1.

To determine the roles of LtgA and LtgB in slow sporulation, we first scraped the wild-type fruiting bodies from CF agar surface (Figure 2C), dispersed spores by sonication, purified their sacculi, and visualized PG using immunofluorescence. Spherical spores from the wild-type cells were visible under DIC microscope, indicating that they retained unflattened shapes. These fruiting body spores lacked PG-specific fluorescence (Figure 4A), consistent with their markedly reduced PG content (Figure 1). However, we cannot exclude the possibility that the polysaccharide spore coats (Voelz and Dworkin, 1962) hinder antibody access to PG. Second, we investigated if *∆ltgA* and *∆ltgB* cells degraded PG during starvation-induced sporulation. Because the aggregates formed by *∆ltgA* and *∆ltgB* cells on CF agar did not contain mature spores, we scrapped cell aggregates from agar surface and purified their sacculi without sonication. The *∆ltgA* cells formed spherical, spore-like cells void of PG (Figure 4A), indicating that while LtgB is sufficient for PG degradation during slow sporulation, LtgA is only required for spore maturation. In contrast, most of the *∆ltgB* cells were still rod-shaped, indistinguishable from vegetative ones (Figure 4A). These observations indicate that LtgB is an essential LTG for PG degradation during slow sporulation.

The two sporulation pathways vary greatly in duration: glycerol-induced rapid sporulation is completed within four hours, while starvation-induced slow sporulation unfolds over several days. Based on this distinction, we hypothesized that LtgA and LtgB could degrade PG at different rates. We used a vanillate-inducible promoter (Iniesta et al., 2012) to overexpress LtgA and LtgB as a merodiploid. Upon induction with 200 μM vanillate, cells overproducing LtgA exhibited heterogeneous morphology, likely resulting from variations in LtgA expression or differences in cell cycle stages within the population. Many cells lost rod shape even in the absence of glycerol, indicating that these cells over-degraded their PG by LtgA (Figure 4B). In contrast, the overexpression of LtgB using the same method did not affect the morphology of vegetative cells (Figure 4B). To test if LtgB overexpression was induced by vanillate, we added a photo-activatable mCherry (PAmCherry) tag to the overexpression constructs of LtgA and LtgB. As shown in Figure 4—figure supplement 1, Figure 4—figure supplement 1—source data 1 and 2 and, in the presence of vanillate, the expression of both LTGs increased significantly. These findings suggest that LtgB exhibits very low LTG activity in vegetative cells, as overexpression of the protein fails to induce substantial PG degradation.

To further investigate the activities of these LTGs, we expressed the periplasmic domains of wild-type LtgA (amino acids 21–243) and LtgB (amino acids 26–710) in *E. coli* (Figure 4C, Figure 4—source data 1). We purified PG from wild-type *M. xanthus* cells, labeled it with Remazol brilliant blue (RBB), and tested if the purified LTGs hydrolyze labeled PG in vitro and release the dye (Jorgenson et al., 2014; Ramirez Carbo et al., 2024; Uehara et al., 2010). Wild-type LtgA solubilized dye-labeled PG, which absorbed light at 595 nm, demonstrating stronger hydrolytic activity than lysozyme, an enzyme that specifically cleaves β–1,4-glycosidic bonds in PG. In contrast, PG incubated with LtgB only showed minimum release of the dye, indicating slow PG hydrolysis (Figure 4C, Figure 4—source data 2). While we cannot rule out the possibility that our purification and reaction conditions were suboptimal for LtgB, both the in vitro RBB assay and the in vivo phenotype resulting from LtgB overexpression support our hypothesis that during vegetative growth, LtgB is less efficient in PG degradation than LtgA.

### LtgB regulates LtgA during glycerol-induced sporulation

Consistent with its role in PG degradation, in a microarray-based transcriptome analysis, *ltgA* transcription was found to increase about twofold during rapid sporulation (4 h) but remain unchanged during slow sporulation (96 h) in the closely related DK1622 strain (Müller et al., 2010). Conversely, *ltgB* expression gradually rises during slow sporulation, reaching 1.8 times the vegetative level at 96 h, while remaining stable during rapid sporulation (Müller et al., 2010; Muñoz-Dorado et al., 2019). However, transcriptomic data alone do not account for the opposing roles of LtgA and LtgB in the rod-to-sphere transition during rapid sporulation. The resemblance in morphology between glycerol-induced *ΔltgB* cells and uninduced cells overproducing LtgA (Figure 4B) suggests that the slower-acting LtgB may offset the rapid activity of LtgA during sporulation, allowing sufficient time for spore maturation.

To test if LtgB regulates LtgA, we constructed a *∆ltgA ∆ltgB* double deletion strain. Cells from this strain shortened their length slightly but failed to abolish rod shape after prolonged glycerol induction, phenocopying the sporulation defect of the *∆ltgA* strain (Figure 2A and B, Figure 2—source data 1). Hence, LtgB is a regulator upstream of LtgA.

### LtgB exhibits a response to glycerol induction earlier than LtgA

Biochemical reactions on PG are unique for the stark size difference between the enzymes and their substrates. While the enzymes are in nanometer scales, their substrates, the PG sacculi, span several micrometers. Under the microscope, PG remains stationary but PG-related enzymes are free to move. Even for *M. xanthus* that moves on surfaces, PG-related enzymes move at least two orders of magnitude faster than the cell/PG (Zhang et al., 2023; Ramirez Carbo et al., 2024). Thus, when diffusive enzymes bind to PG, their mobility decreases (Lee et al., 2016; Zhang et al., 2023). For instance, DacB, another PG hydrolase, reduces its single-particle mobility in the conditions where its activity is activated (Zhang et al., 2023). By tracking single fluorescently labeled enzyme particles, we can approximate their PG-binding in different physiological conditions and genetic backgrounds (Ramirez Carbo et al., 2024; Zhang et al., 2023; Ramirez Carbo and Nan, 2026). We individually expressed PAmCherry-labeled LtgA and LtgB using their native loci and promoters. Both labeled LTGs accumulated as full-length proteins (Figure 5—figure supplement 1A, Figure 5—figure supplement 1—source data 1) and the PAmCherry tags did not negatively affect either glycerol or starvation-induced sporulation pathways, indicating that these fusion proteins were fully functional (Figure 5—figure supplement 1B and C, Figure 5—figure supplement 1—source data 2 and 3). Notably, vegetative cells produce significantly less LtgA than LtgB, consistent with the RNAseq data in which *ltgA* transcription was below the detection limit (Muñoz-Dorado et al., 2019; Figure 5—figure supplement 1A).

We used a 405 nm excitation laser (0.3 kW/cm^2^, 0.1 s) to activate the fluorescence of a few labeled LTG particles randomly in each cell and quantified their localization using a 561 nm laser at 10 Hz using single particle tracking photo-activated localization microscopy (sptPALM, see ‘Materials and methods’; Fu et al., 2018; Nan et al., 2015; Nan et al., 2013; Ramirez Carbo and Nan, 2026). As free PAmCherry particles diffuse extremely fast in the cytoplasm, entering and exiting the focal plane frequently, they usually appear as blurry objects that cannot be followed at 10 Hz close to the cell surface (Fu et al., 2018; Zhang et al., 2023). For this reason, the noise from free PAmCherry due to potential protein degradation was negligible.

**Figure 5.**
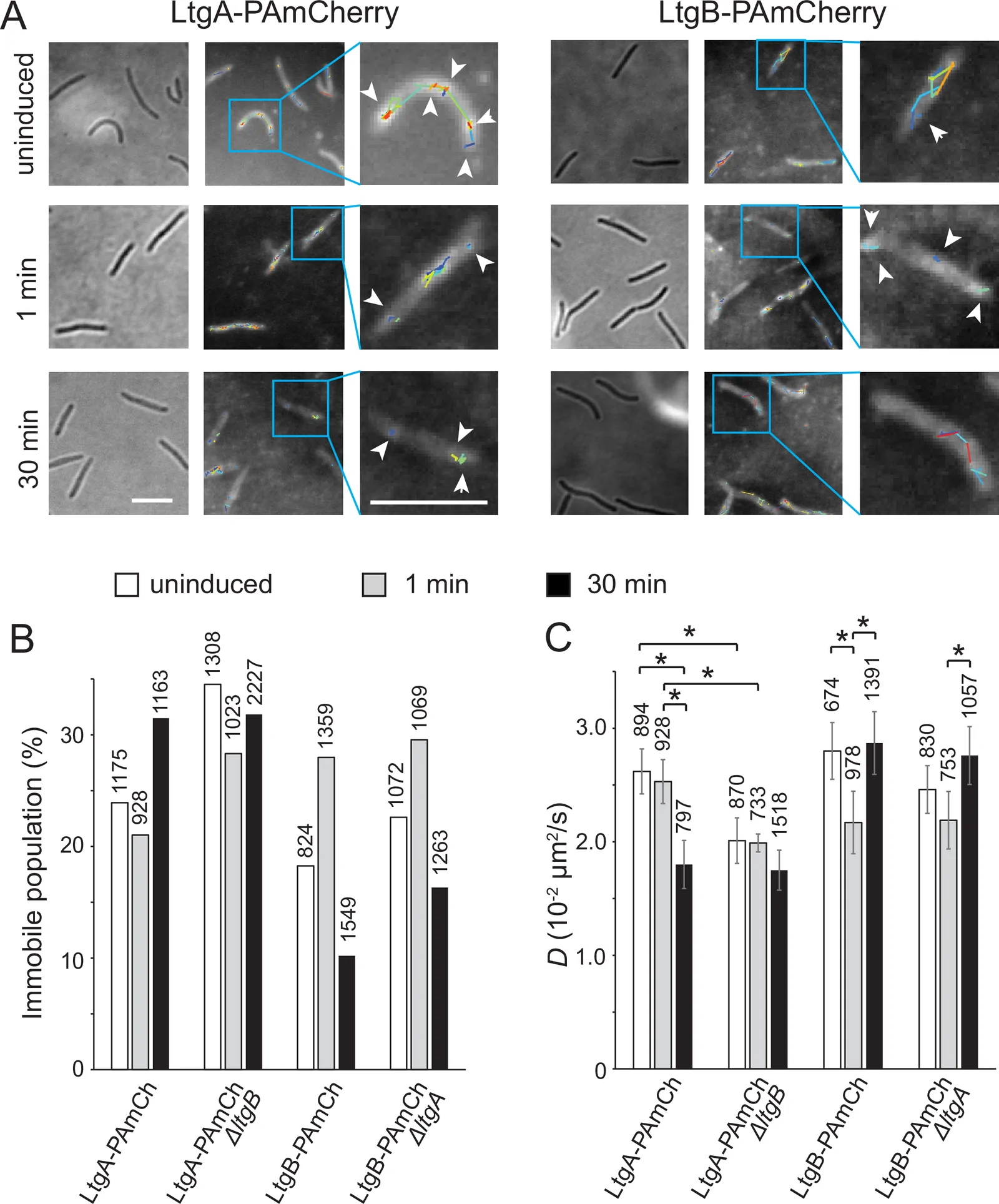
LtgB regulates the PG-binding of LtgA during glycerol-induced sporulation. (**A**) LtgB exhibits a response to glycerol induction earlier than LtgA. Representative trajectories of LtgA and LtgB before (uninduced) and after (1 min and 30 min) glycerol induction. The overall distribution of both LTGs is displayed using the composite of 100 consecutive frames taken at 100 ms intervals. Single-particle trajectories of PAmCherry were generated from the same frames. Individual trajectories are distinguished by colors. Scale bars, 5 μm. (**B, C**) The absence of LtgB reduces the diffusion of LtgA, which is reflected in the increase in immobile population (**B**) and the decrease in diffusion coefficient (*D*) (**C**), and these effects are especially prominent in the cells before (uninduced) and immediately after (1 min) glycerol induction. For each protein and condition, particles were identified from at least 100 cells and three independent experiments. The total number of particles analyzed is shown on top of each plot. Error bars were the standard deviation of 1000 bootstrap samples, and * indicates a difference of >0.005. Figure 5—source data 1.Diffusion coefficient data for Figure 5C.

Single particles of PAmCherry-labeled LtgA and LtgB can be categorized into two populations, immobile and mobile. The immobile particles remained within a single pixel (160 nm × 160 nm) before photobleach, and the mobile ones displayed typical diffusion (Figure 5A, Figure 5—source data 1). For the particles that switched between mobile and immobile states, our algorithm categorized them as mobile and calculated their diffusion coefficients (*D*) from their entire trajectories that contained both mobile and immobile segments. Hence, binding to PG not only increases the immobile population of the enzyme particles but also decreases the *D* of the mobile particles. In vegetative cells where large-scale PG degradation does not occur, 23.9% (n=1175) and 18.2% (n=824) of LtgA and LtgB particles were immobile, respectively (Figure 5B, Figure 5—source data 1). *D* values of the mobile population were (2.62±0.20)×10^–2^ μm^2^/s (n=894) for LtgA-PAmCherry and (2.80±0.25)×10^–2^ μm^2^/s (n=674) for LtgB-PAmCherry (Figure 5C, Figure 5—source data 1).

**Figure 5—figure supplement 1.**
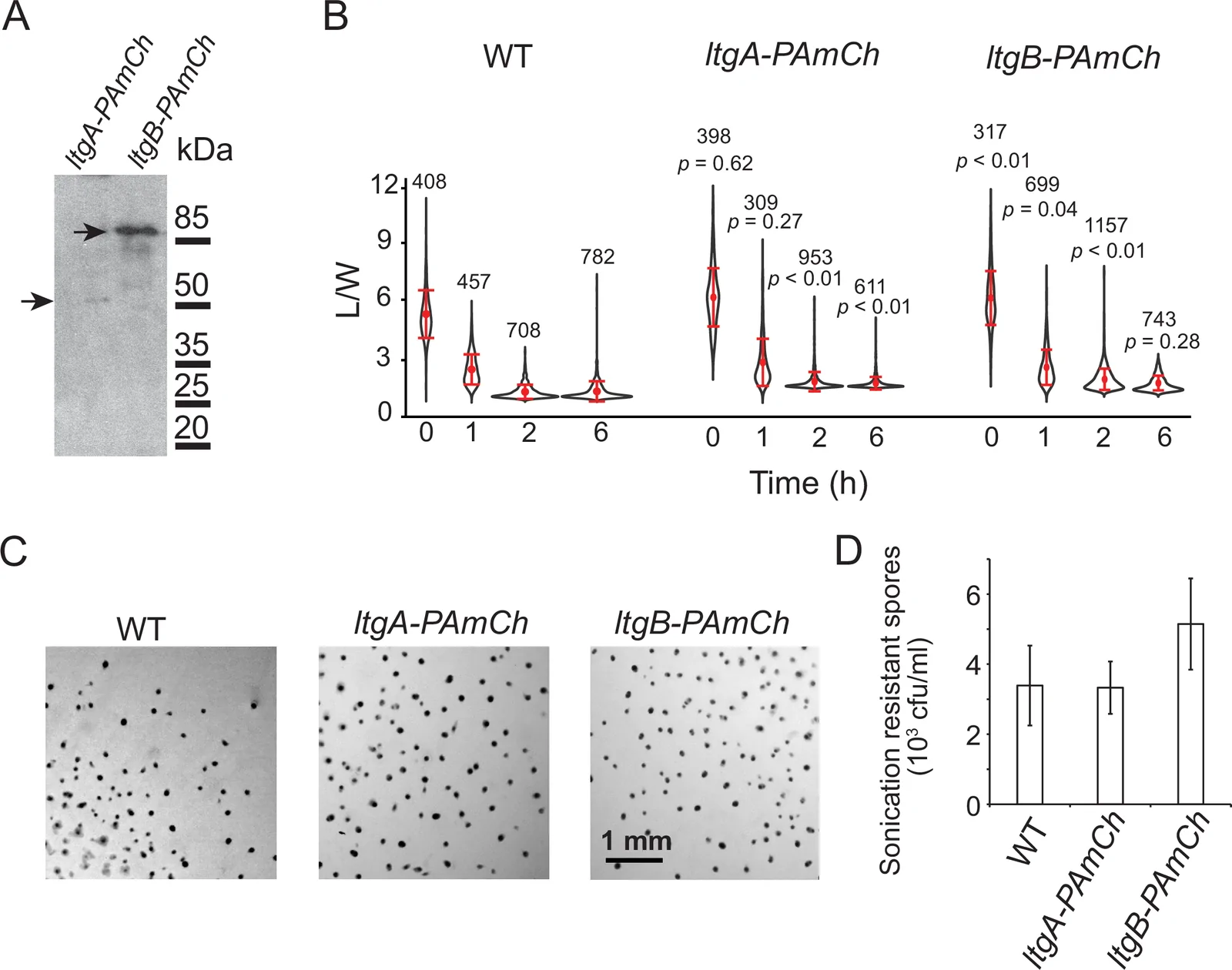
PAmCherry-labeled LtgA and LtgB express as full-length and fully functional proteins. (**A**) PAmCherry-labeled LtgA and LtgB are expressed as full-length proteins in vegetative cells (53 kDa and 103 kDa, respectively), which were detected by an anti-mCherry antibody. (**B**) The PAmCherry labels do not affect glycerol-induced sporulation. Quantitative analysis of glycerol-induced sporulation using the length/width ratio (L/W) of cells. Whiskers indicate the 25th–75th percentiles and red dots the median. The total number of cells analyzed is shown on top of each plot. p values were calculated against the wild-type data at the same time points using a single-tailed ANOVA test on unweighted samples. (**C**) The PAmCherry labels do not inhibit starvation-induced fruiting body formation on agar surfaces. The images were taken after 96 h of incubation on CF agar. (**D**) The PAmCherry labels do not inhibit the formation of starvation-induced spores. Sonication-resistant spores were counted as colony formation units (cfu) after 96 h of incubation on CYE agar. Data are shown as mean ± standard deviation (n=3). Figure 5—figure supplement 1—source data 1.Unlabeled image of Figure 5—figure supplement 1A. Figure 5—figure supplement 1—source data 2.Cell morphology measurements for Figure 5—figure supplement 1B. Figure 5—figure supplement 1—source data 3.Spore count for Figure 5—figure supplement 1C.

We then determined how the dynamics of both LTGs respond to glycerol induction in the rapid sporulation pathway. Immediately (1 min) after adding glycerol, the dynamics of LtgA remained little changed, when 21.0% (n=928) of particles were immobile and the *D* of the mobile ones was (2.53±0.19)×10^–2^ μm^2^/s (n=733) (Figure 5B and C, Figure 5—source data 1). In contrast, the mobility of LtgB decreased significantly, with the immobile population increased to 28.0% (n=1359) and *D* of the mobile population decreased to (2.17±0.28)×10^–2^ μm^2^/s (n=978) (Figure 5B and C, Figure 5—source data 1). Therefore, these results suggest that upon glycerol induction, LtgB rapidly enhances its binding to PG.

We then chose 30 min after glycerol induction as a time point for rapid PG degradation, which is reflected by the dramatic decrease of L/W during the first hour of sporulation (Figure 2A and B, Figure 2—source data 1). Compared to its inert response 1 min after glycerol induction, the mobility of LtgA particles decreased significantly at 30 min, when the immobile population increased to 31.5% (n=1163) and *D* of the mobile population decreased to (1.80±0.21)×10^–2^ μm^2^/s (n=797) (Figure 5B and C, Figure 5—source data 1). The reduced mobility of LtgA aligns with its function as the primary LTG in glycerol-induced sporulation. Strikingly different from LtgA, as sporulation advanced to the 30 min mark, LtgB’s mobility returned to its pre-induction level, with the immobile population decreased to 10.2% (n=1549) and *D* of the mobile population increased to (2.87±0.28)×10^–2^ μm^2^/s (n=1391) (Figure 5B and C, Figure 5—source data 1). Therefore, the PG-binding of LtgB shows a negative correlation with PG degradation. Taken together, LtgA and LtgB display opposite responses to glycerol induction, in which LtgB rapidly binds to PG before yielding to LtgA, whose PG-binding is concurrent with the rod-to-sphere transition.

### LtgB blocks LtgA from binding PG in the early stage of glycerol-induced sporulation

Does LtgB’s early PG-binding suppress PG degradation by LtgA? To answer this question, we expressed LtgA-PAmCherry using the native *ltgA* locus and promoter in the *ΔltgB* background. In vegetative cells, the absence of LtgB significantly reduced the mobility of single LtgA-PAmCherry particles, suggesting that LtgB does affect LtgA’s binding to the PG (Figure 5B and C, Figure 5—source data 1). Similarly, we expressed LtgB-PAmCherry using the native *ltgB* locus and promoter in the *ΔltgA* background. In contrast, the mobility of single LtgB-PAmCherry particles only decreased slightly in the absence of LtgA (Figure 5B and C, Figure 5—source data 1). These results suggest that while LtgB significantly reduces LtgA’s binding to PG, probably due to its higher expression level, LtgA has only a modest impact on LtgB’s ability to bind PG.

Strikingly different from its slow response to glycerol in wild-type cells, LtgA increased its PG-binding immediately (1 min) after glycerol induction in the *ΔltgB* background, confirming that LtgB may compete with LtgA for access to PG in the early stage of sporulation (Figure 5B and C, Figure 5—source data 1). In contrast, at 30 min of induction, a time point when LtgB dissociated from PG, its absence no longer affected LtgA’s PG-binding (Figure 5B and C, Figure 5—source data 1). Similar to the observation in wild-type cells, the lack of LtgA did not affect the PG-binding of LtgB at either 1 min or 30 min after glycerol induction (Figure 5B and C, Figure 5—source data 1). In summary, the sequential PG-binding by these two LTGs controls the pace of PG degradation during glycerol-induced sporulation. LtgB, a slow LTG, regulates the progression of rapid sporulation by preventing LtgA, a fast LTG, from excessively degrading PG, thereby ensuring spore maturation.

### Upregulated PG synthesis negatively affects PG degradation

Since LtgA is less mobile in vegetative *ΔltgB* cells, that is, strongly binds to PG (Figure 5B and C, Figure 5—source data 1), why do these cells retain their rod shape rather than losing it spontaneously before glycerol induction? In addition to PG-binding, LtgA may require additional stimuli to initiate PG degradation. We hypothesized that, similar to certain antibiotics targeting PG synthases, which induce wild-type cells to degrade PG and form pseudospores in rich liquid media (Zhang et al., 2023; O’Connor and Zusman, 1997), glycerol may alter cellular metabolism, leading to reduced PG synthesis. If this is the case, cells with upregulated PG synthesis should remain rods even in the presence of glycerol.

To test our hypothesis, we used the vanillate-inducible promoter (Iniesta et al., 2012) to overexpress *murA* as a merodiploid in the wild-type background. Because MurA catalyzes the first committed step of PG synthesis that produces UDP-MurNAc (Egan et al., 2020; Rohs and Bernhardt, 2021), elevated MurA levels are expected to channel more cellular resources toward PG synthesis. Overproduction of MurA in the presence of 200 μM vanillate resulted in a heterogeneous cell population, with normal cells coexisting alongside elongated ones (Figure 2A and B, Figure 2—figure supplement 2, Figure 2—figure supplement 2—source data 1). Similar to the cells that overexpressed LtgA (Figure 4B), this heterogeneity likely reflects variable *murA* induction or the unsynchronized growth stages within the population. Overall, excessive MurA increased the average length of vegetative cells by 19.6%. Cells overproducing MurA also displayed heterogeneity during glycerol-induced sporulation. While some cells transitioned into spheres, many retained their rod shape even after 6 h of induction (Figure 2A and B, Figure 2—source data 1). Notably, sporadic lysis of rod-shaped cells began 2 h post-induction and became increasingly frequent with continued incubation (Figure 2A). Potentially, elevated muropeptide production may lead to the accumulation of toxic intermediates that the relatively insufficient LTGs failed to degrade (Weaver et al., 2022). Nevertheless, the capacity of MurA overexpression to enable certain cells to circumvent glycerol-induced sporulation implies that PG production acts as a regulatory cue for PG degradation.

To test if excessive MurA can also reduce PG degradation during slow sporulation, we induced MurA overexpression on solid CF agar containing 100 μM vanillate. After 96 h of incubation, these cells developed fruiting body-like aggregates (Figure 3A). To test if such fruiting bodies contained mature spores, we scraped them from submerged CF medium and tested the germination rate of spores on CYE agar after sonication. The fruiting bodies formed by cells overexpressing MurA yielded 7.6% of the colonies produced by an equivalent number of wild-type cells, indicating that upregulated PG synthesis also prevents slow sporulation. In contrast, cells grown without vanillate progressed normally through glycerol-induced sporulation and starvation-induced fruiting body formation, showing no differences compared to wild-type cells (Figure 3B). Therefore, the sporulation of *M. xanthus* is sensitive to PG synthesis and increased PG synthesis balances PG degradation in both sporulation pathways.

## Discussion

Our findings demonstrate that *M. xanthus*, a non-firmicute bacterium, relies on PG degradation to change cell shape during sporulation. This mechanism presents a significant challenge for sporulating cells: preserving their structural integrity while simultaneously dismantling the primary framework that upholds it. Our findings revealed that besides regulating the production of LTGs, cells could control the pace of PG degradation in a rapid sporulation pathway through the regulation between two LTGs and hence allow spore maturation.

Studying the regulation between two LTGs is especially challenging both in vivo and in vitro for a few reasons. First, it is difficult to monitor their instantaneous activities in vivo. Secondly, muropeptide analysis may not distinguish their roles because their products bear the same signature, anhydro-MurNAc. Thirdly, some enzymes require special substrates or activators, which would be difficult to study in vitro. To overcome these technical hurdles, we leveraged single-particle mobility to quantify the PG-binding of these enzymes. The simultaneous occurrence of reduced LtgA mobility and PG degradation during glycerol-induced sporulation indicates that the molecular dynamics of LtgA accurately mirrors its enzymatic activity. Such correlation between decreased particle mobility and increased enzymatic activity applies to many other PG-related enzymes, including multiple PG polymerases in *E. coli* and the endopeptidase DacB in *M. xanthus* (Lee et al., 2016; Zhang et al., 2023; Yang et al., 2021). Because the size difference between PG and its related enzymes is a conserved feature in all bacteria, our method using single-particle tracking to quantify PG-binding can be applied in many organisms.

We discovered that LtgB, a slower-acting LTG, limits the access of the faster LtgA to the PG during the early phase of rapid sporulation, thereby regulating the rate of its degradation. Then how does LtgA breach the blockage of LtgB and gain access to PG as sporulation progresses? Because the overexpression of LtgA is sufficient to damage PG in uninduced cells (Figure 4B), we propose that the relative abundance of these two LTGs plays a critical role in determining the fate during fast sporulation. While LtgB nearly saturates PG binding sites in vegetative cells where few LtgA are produced, LtgA—upregulated after glycerol induction (Müller et al., 2010) —gradually outcompetes LtgB and thereby causes LtgB to disassociate from PG.

Although glycerol has been known for decades to trigger sporulation in *M. xanthus*, the underlying mechanism remains elusive. Notably, glycerol was recently found to disrupt PG synthesis in *B. subtilis*; in contrast, this effect inhibits *B. subtilis* sporulation, which depends on enhanced PG synthesis (Updegrove et al., 2026). While elevated glycerol concentrations are associated with reduced PG synthesis in both species, whether and how glycerol affects PG synthesis in *M. xanthus* remains unclear. Furthermore, it is unknown whether this apparent correlation reflects a shared evolutionary origin or a common mechanistic basis. Nevertheless, PG dynamics must play critical roles in bacterial sporulation.

As increased PG synthesis enables many *M. xanthus* cells to bypass both sporulation pathways, it is reasonable to propose that diminished PG synthesis initiates PG degradation by LTGs. While functional coordination between PG synthesis and hydrolysis has been speculated for over 50 years (Koch, 1985; Koch, 1990), its mechanisms only came to light recently. Some endopeptidases are proposed to serve as the space-makers for PG synthases (Dörr et al., 2013; Singh et al., 2012). In *M. xanthus*, we reported that moenomycin, an antibiotic that specifically binds to the GTase domains of class A penicillin-binding proteins (aPBPs) (Sung et al., 2009; Lovering et al., 2007), specifically activates a PG endopeptidase DacB through PBP1a2, an aPBP (Zhang et al., 2023). Because moenomycin mimics a growing glycan strand in the GTase domains of aPBPs, it actually locks aPBPs in their glycan-charged conformations (Sung et al., 2009; Lovering et al., 2007). Thus, similar to the moenomycin-bound form, glycan-charged PBP1a2 in physiological conditions recruits DacB to the PG assembly sites, which, in turn, generates openings in the existing PG network as the crosslink sites for the TPase activity of PBP1a2. Such ‘make-before-break’ mechanism (Koch and Doyle, 1985) could prevent unneeded hydrolysis and thus maintain PG integrity.

Compared to the well-characterized space-making functions of endopeptidases, the roles of LTGs, beyond PG recycling, remain ill-defined. Even less is known about their potential coordination with PG synthases (Weaver et al., 2023). Because LTGs from bacterial and phage origins are inhibited by L,D-crosslinks, they may functionally connect to PBPs that form D,D-crosslinks (Alvarez et al., 2024). Additionally, some LTGs, such as MltD and MltG in *E. coli* and MltG in *Vibrio cholerae*, prefer to degrade nascent, uncrosslinked glycan strands (Kaul et al., 2024; Weaver et al., 2022; Bohrhunter et al., 2021). Our recent report indicates that AgmT, an *M. xanthus* LTG homologous to *E. coli* MltG, detoxifies uncrosslinked glycan strands under antibiotic stress and modifies crosslinked PG scaffolds to attach a motility machinery to PG (Ramirez Carbo et al., 2024). Then how does reduced PG synthesis activate LtgA in *M. xanthus*? Although MltE, the closest homolog of LtgA in *E. coli*, degrades glycan strands regardless of their crosslinking status or peptide content, it shows a marked preference for uncrosslinked substrates (Dik et al., 2017; Fibriansah et al., 2012). Similar to AgmT and MltE, LtgA might also bind to both crosslinked and uncrosslinked glycan strands. Consistent with its high mobility, LtgA may coordinate with diffusive PG polymerases and cleave the uncrosslinked glycan strands. During rapid sporulation, as nascent glycan strands decline due to muropeptide depletion, LtgA increasingly binds to the crosslinked PG scaffold, which is evidenced by its reduced mobility. This binding intensifies further as PG degradation continues, establishing a positive feedback loop. In this case, LtgB serves as a brake that dampens this positive feedback, moderates PG degradation, and thus allows sporulating cells to maintain integrity by assembling polysaccharide coats. Such cross-regulation between PG hydrolases, which is currently under investigation, provides yet another mechanism by which bacteria dynamically maintain their PG cell walls.

## Materials and methods

**Key resources table:** 

Reagent type (species) or resource	Designation	Source or reference	Identifiers	Additional information
Gene (*Myxococcus xanthus*)	*ltgA* (K1515_20820)	Aramayo and Nan, 2022	MXAN_RS16290 MXAN_3363	Slt/MltE LTG family 1
Gene (*M. xanthus*)	*ltgB* (K1515_17460)	Aramayo and Nan, 2022	MXAN_RS19615 MXAN_4034	Slt/MltE LTG family 1
Gene (*M. xanthus*)	*murA* (K1515_13170)	Aramayo and Nan, 2022	MXAN_RS28325 MXAN_4909	
Gene (*M. xanthus*)	*agmT*	Aramayo and Nan, 2022 Ramirez Carbo et al., 2024	K1515_04910 MXAN_6607	MltG/YceG LTG family 5
Gene (*M. xanthus*)	K1515_37860	Aramayo and Nan, 2022	MXAN_RS00570 MXAN_0114	Slt/MltE LTG family 1
Gene (*M. xanthus*)	K1515_37385	Aramayo and Nan, 2022	MXAN_RS01035 MXAN_0210	Slt/MltE LTG family 1
Gene (*M. xanthus*)	K1515_34725	Aramayo and Nan, 2022	MXAN_RS03640 MXAN_0754	LTG family unknown
Gene (*M. xanthus*)	K1515_24775	Aramayo and Nan, 2022	MXAN_RS12365 MXAN_2558	RlpA LTG family 6
Gene (*M. xanthus*)	K1515_22185	Aramayo and Nan, 2022	MXAN_RS14935 MXAN_3081	Slt/MltE LTG family 1
Gene (*M. xanthus*)	K1515_20905	Aramayo and Nan, 2022	MXAN_RS16205 MXAN_3344	Slt/MltE LTG family 1
Gene (*M. xanthus*)	K1515_14545	Aramayo and Nan, 2022	MXAN_RS22465 MXAN_4628	Slt/MltE LTG family 1
Gene (*M. xanthus*)	K1515_09355	Aramayo and Nan, 2022	MXAN_RS27580 MXAN_5690	Slt/MltD/MltE, LTG family 1
Gene (*M. xanthus*)	K1515_06035	Aramayo and Nan, 2022	MXAN_RS30855 MXAN_6370	Slt/MltE LTG family 1
Gene (*M. xanthus*)	K1515_01490	Aramayo and Nan, 2022	MXAN_RS35365 MXAN_7308	MltA LTG family 2
Gene (*M. xanthus*)	K1515_01440	Aramayo and Nan, 2022	MXAN_RS35415 MXAN_7318	Slt/MltD/MltE, LTG family 1
Strain, strain background (*M. xanthus*)	DZ2	Campos and Zusman, 1975	DZ2	Wild-type strain
Strain, strain background (*M. xanthus*)	*ΔltgA*	This paper	BN361	Request from Nan lab
Strain, strain background (*M. xanthus*)	*ΔltgB*	This paper	BN362	Request from Nan lab
Strain, strain background (*M. xanthus*)	*ΔltgA ΔltgB*	This paper	BN363	Request from Nan lab
Strain, strain background (*M. xanthus*)	*ΔltgA attB::P_van_-ltgB*	This paper	BN364	Request from Nan lab
Strain, strain background (*M. xanthus*)	*attB::P_van_-ltgA*	This paper	BN365	Request from Nan lab
Strain, strain background (*M. xanthus*)	*attB::P_van_-ltgB*	This paper	BN366	Request from Nan lab
Strain, strain background (*M. xanthus*)	*attB::P_van_-ltgA-PAmCherry*	This paper	BN367	Request from Nan lab
Strain, strain background (*M. xanthus*)	*attB::P_van_-ltgB-PAmCherry*	This paper	BN368	Request from Nan lab
Strain, strain background (*M. xanthus*)	*attB::P_van_-murA*	This paper	BN369	Request from Nan lab
Strain, strain background (*M. xanthus*)	*ltgA-PAmCherry*	This paper	BN370	Request from Nan lab
Strain, strain background (*M. xanthus*)	*ltgB-PAmCherry*	This paper	BN371	Request from Nan lab
Strain, strain background (*M. xanthus*)	*ΔltgB ltgA-PAmCherry*	This paper	BN372	Request from Nan lab
Strain, strain background (*M. xanthus*)	*ΔltgA ltgB-PAmCherry*	This paper	BN373	Request from Nan lab
Strain, strain background (*M. xanthus*)	*K1515_37860::kan*	This paper	BN374	Request from Nan lab
Strain, strain background (*M. xanthus*)	*K1515_37385::kan*	This paper	BN375	Request from Nan lab
Strain, strain background (*M. xanthus*)	*K1515_34725::kan*	This paper	BN376	Request from Nan lab
Strain, strain background (*M. xanthus*)	*K1515_24775::kan*	This paper	BN377	Request from Nan lab
Strain, strain background (*M. xanthus*)	*K1515_22185::kan*	This paper	BN378	Request from Nan lab
Strain, strain background (*M. xanthus*)	*K1515_20905::kan*	This paper	BN379	Request from Nan lab
Strain, strain background (*M. xanthus*)	*K1515_14545::kan*	This paper	BN380	Request from Nan lab
Strain, strain background (*M. xanthus*)	*K1515_09355::kan*	This paper	BN381	Request from Nan lab
Strain, strain background (*M. xanthus*)	*K1515_06035::kan*	This paper	BN382	Request from Nan lab
Strain, strain background (*M. xanthus*)	*K1515_01490::kan*	This paper	BN383	Request from Nan lab
Strain, strain background (*M. xanthus*)	*K1515_01440::kan*	This paper	BN384	Request from Nan lab
Strain, strain background (*Escherichia coli*)	DH5α	Lab collection		
Strain, strain background (*E. coli*)	BL31 (DE3)	Lab collection		
Antibody	Rabbit polyclonal anti-mCherry	Rockland Immunochemicals	600-401-P16	(1:2000)
Antibody	Rabbit polyclonal anti-MreB	Mauriello et al., 2010		(1:20,000)
Antibody	Goat polyclonal anti-rabbit IgG H+L (HRP)	Fisher Scientific	31460	(1:20,000)
Software, algorithm	MATLAB	MathWorks		
Software, algorithm	Single-particle analysis	Fu et al., 2018; Zhang et al., 2023; Ramirez Carbo and Nan, 2026		https://doi.org/10.5281/zenodo.8234126
Software, algorithm	ImageJ	https://imagej.net		

### Bacterial strains and growth conditions

Vegetative *M. xanthus* cells were grown in liquid CYE medium (10 mM MOPS pH 7.6, 1% [w/v] Bacto casitone [BD Biosciences], 0.5% yeast extract, and 8 mM MgSO_4_) at 32°C, in 125 ml flasks with vigorous shaking, or on CYE plates that contains 1.5% agar. We used strain DZ2 as the wild-type *M. xanthus* strain (Campos and Zusman, 1975). Knock-out mutants were constructed by electroporating DZ2 cells with 4 μg of plasmid DNA. Transformed cells were plated on CYE plates supplemented with 100 mg/ml sodium kanamycin sulfate or 10 mg/ml tetracycline. The strains used in this study are listed in the Key Resources Table and plasmids and primers in Supplementary file 1. All strains and plasmids created in this study are available upon request to BN.

### Sporulation and spore purification

Cells were grown in 25 ml liquid cell culture to OD_600_ 0.8–1.2. Glycerol was added to 1 M to induce sporulation. The liquid culture was incubated at 32°C with vigorous shaking. After 24 h, spores and cells were harvested by centrifugation (10 min, 10,000 × *g,* 25°C). To further purify the wild-type spores, the pellet was resuspended in 5 ml water, the remaining vegetative cells were eliminated by sonication (Cole-Palmer 4710 ultrasonic homogenizer, 30% output, 10 cycles), and sonication-resistant spores were washed three times with water and collected by centrifugation (5 min, 10,000 × *g,* 4°C). The elimination of vegetative cells was confirmed by DIC microscopy.

For phenotypic assays on starvation-induced fruiting body formation, vegetative cells (10 ul), at a concentration of 4×10^9^ ml^–1^, were spotted on CF (0.015% Casitone, 0.2% sodium citrate, 0.1% sodium pyruvate, 0.02% (NH_4_)_2_SO_4_, 10 mM MOPS [pH 7.6], 8 mM MgSO_4_ and 1 mM KH_2_PO_4_) plates containing an agar concentration of 1.5%, incubated at 32°C. To purify fruiting body spores, cells were grown in 25 ml liquid cell culture to OD_600_ 0.8–1.2 and harvested by centrifugation (10 min, 10,000 × *g,* 25 °C). The pellet was resuspended in 1 ml water and plated on two 150 mm CF plates containing 1.5% agar using a spreader. The plates were air-dried and incubated at 32°C for 120 h. For the wild-type and *∆ltgA* strains, fruiting bodies were scraped from the plates, suspended in 2 ml water, and subjected to sonication (Cole-Palmer 4710 ultrasonic homogenizer, 60% output, 20 cycles). Spores were washed three times in water and collected by centrifugation (5 min, 10,000 × *g,* 4°C). For the *∆ltgB* strains, the sonication step was omitted.

### Quantification of starvation-induced spores

We used the submerged starvation culture to quantify starvation-induced spores. Cells were first grown in liquid CYE medium overnight and adjusted to OD_600_ 1 using fresh CYE. 50 ml of the adjusted culture was then poured into a Ø 150 mm polystyrene Petri dish and incubated without shaking for 24 h at 32°C. Liquid and unattached cells were poured out carefully before adding 50 ml liquid CF medium. Petri dishes were incubated without shaking for 96 h at 32°C. Finally, liquid and unattached cells were poured out carefully and the cells attached to the bottom of Petri dishes were scraped and resuspened into 1 ml water. The suspensions were then sonicated to eliminate vegetative cells, diluted and plated on solid CYE medium. Sonication-resistant spores were then numerated by counting the colonies after a 96 h incubation at 32°C. Data were collected using three biological replicates.

### Peptidoglycan purification and UPLC analysis

For PG analysis, samples were processed as previously described for Gram-negative bacteria (Alvarez et al., 2016; Desmarais et al., 2013). Vegetative cells were harvested at mid-stationary phase by centrifugation (30 min, 8000 × *g*). Vegetative cells and purified spores (as described in the previous section) were resuspended and boiled in 1x phosphate-buffered saline (PBS) with 5% SDS for 2 h. The sacculi were repeatedly washed with MilliQ water by ultracentrifugation (150,000 × *g*, 10 min, 20°C) to remove the remaining SDS. The samples were then treated with muramidase (100 μg/ml) for 16 h at 37°C. Muramidase digestion was stopped by boiling and coagulated proteins were removed by centrifugation (10 min, 15,000 × *g*). The supernatants were first adjusted to pH 8.5–9.0 with sodium borate buffer and then sodium borohydride was added to a final concentration of 10 mg/ml. After reducing the samples at room temperature for 30 min, the pH was adjusted to pH 3.5 with orthophosphoric acid.

UPLC analyses of muropeptides were performed on a Waters UPLC system (Waters Corp., USA) equipped with an ACQUITY UPLC BEH C18 Column (Waters Corp.) and a dual-wavelength absorbance detector. Elution of muropeptides was detected at 204 nm. Muropeptides were separated at 45°C using a linear gradient from buffer A (0.1% formic acid in water) to buffer B (0.1% formic acid in acetonitrile) in a 25 min run, with a 0.30 ml/min flow.

### Muropeptide identification

Muropeptide identity was confirmed by MS and MS/MS analysis on a Xevo G2/XS Q-TOF mass spectrometer (Waters Corp.) coupled to the UPLC system with the same column, solvents, and gradients as mentioned above. Data acquisition and processing were performed using the UNIFI software package (Waters Corp.). Muropeptides were assigned based on (i) accurate mass matching to theoretical monoisotopic masses of expected *M. xanthus* PG building blocks (Bui et al., 2009; White et al., 1968) and (ii) comparison of retention times with those reported in previous analysis with similar PG compositions. For vegetative cells analysis, relative total PG amounts were calculated by comparison of the total intensities of the chromatograms (total area) from three biological replicas normalized to the same initial biomass and extracted with the same volumes. Quantification of muropeptides was based on their relative abundances (relative area of the corresponding peak).

### Imaging and data analysis

For all imaging experiments on isolated spores/cells, we spotted 5 μl of cells grown in liquid CYE medium to OD_600_~1 on agar (1.5%) pads and imaged using an Andor iXon Ultra 897 EMCCD camera (effective pixel size 160 nm) on an inverted Nikon Eclipse-Ti microscope with a 100× 1.49 NA TIRF objective. Fruiting bodies were photographed after a 120 h incubation on CF agar using a Nikon SMZ1000 microscope and an OMAX A3590U digital camera. Cell morphology was imaged at indicated time points using DIC microscopy. The geometric aspect ratios (L/W) of spores/cells were determined using a custom algorithm written in MATLAB, which is available in the GitHub repository, https://github.com/NanLabMyxo/Rod_shape_paper copy archived at NanLabMyxo, 2026 (Zhang et al., 2020; Zhang et al., 2023).

For sptPALM, *M. xanthus* cells were grown in CYE to 4×10^8^ ml, spotted on 1.5% agar pads and subjected to highly inclined and laminated optical sheet illumination (Fu et al., 2018; Nan et al., 2015; Nan et al., 2013; Tokunaga et al., 2008). PAmCherry was activated using a 405 nm laser (0.3 kW/cm^2^), excited, and imaged using a 561 nm laser (0.2 kW/cm^2^). Images were acquired at 10 Hz. For each sptPALM experiment, single PAmCherry particles were localized in at least 100 individual cells from three biological replicates. sptPALM data were analyzed using a MATLAB (MathWorks) script, which is available in the GitHub repository, https://github.com/NanLabMyxo/Rod_shape_paper copy archived at NanLabMyxo, 2026 (Zhang et al., 2020; Zhang et al., 2023; Ramirez Carbo and Nan, 2026). Briefly, cells were identified using DIC images. Single PAmCherry particles inside cells were fit by a symmetric 2D Gaussian function, whose center was assumed to be the particle’s position (Fu et al., 2018). Particles that explored areas smaller than 160 nm × 160 nm (within one pixel) in 0.4–1.2 s were considered immotile (Fu et al., 2018; Zhang et al., 2023). *D* of all the mobile particles was determined from a linear fit to the first four points of the MSD using the formula MSD = 4*DΔt* (Fu et al., 2018; Lee et al., 2016). Error bars were the standard deviation of 1000 bootstrap samples using the published method (Morgenstein et al., 2015). Sample trajectories were generated using the TrackMate (Ershov et al., 2022) plugin in the ImageJ suite (https://imagej.net).

### Experimental study design

Cell morphology data and single-particle data were pooled from three independent experiments on biological replicates. The number of data points is available in the figures. Cell size and single-particle data were analyzed using our algorithm (https://www.pnas.org/cgi/doi/10.1073/pnas.2001384117 and https://github.com/NanLabMyxo/Rod_shape_paper copy archived at NanLabMyxo, 2026) without human intervention. To further eliminate potential bias, two researchers ran analysis algorithms independently, one of them doing so in a blind fashion. Muropeptide analysis was performed once on three biological replicates. No data were excluded.

### Immunofluorescence

Cell samples were resuspended and boiled in 1× PBS with 5% SDS for 2 h. The sacculi were repeatedly washed with water by centrifugation (5 min, 15,000 × *g,* 25°C) to remove the remaining SDS. Remaining proteins were further removed by incubation with proteinase K (1 µg/ml) for 2 h at 37°C. Microscope cover slides were prepared by coating with 100 µl 0.1% poly-L-lysine for 5 min at room temperature (RT), washing with ddH_2_O and air drying. 100 µl samples were spotted onto the slides and incubated for 20 min at RT. The slides were then washed three times with PBS (81 mM Na_2_HPO_4_, 15 mM KH_2_PO_4_, 1.37 M NaCl, 27 mM KCl, pH 7.4), and then incubated with anti-PG primary antibody^16^ diluted 1:1000 in PBS at RT for 1 h. Slides were washed six times with PBS and then incubated with Alexa Fluor 488 conjugated goat-anti-rabbit secondary antibody (Invitrogen) at a 1:1000 dilution in PBS at RT for 1 h in the dark. Slides were washed six times with PBS. Each cover glass was applied to a supporting slide, fixed with nail polish, and stored at 4°C, if necessary. Images were recorded using a 488 nm laser.

### Protein expression and purification

DNA sequences encoding amino acids 21–243 of LtgA and 26–710 of LtgB were amplified by polymerase chain reaction (PCR) and inserted into the pET28a vector (Novagen) between the restriction sites of *Eco*RI and *Hind*III and used to transform *E. coli* strain BL21(DE3). Transformed cells were cultured in 20 ml LB (Luria-Bertani) broth at 37°C overnight and used to inoculate 1 l LB medium supplemented with 1.0% glucose. Protein expression was induced by 0.1 mM IPTG (isopropyl-h-d-thiogalactopyranoside) when the culture reached an OD_600_ of 0.8. Cultivation was continued at 16°C for 10 h before the cells were harvested by centrifugation at 6000×*g* for 20 min and lysed by sonication in buffer A (20 mM Tris-HCl pH 8.0, 200 mM NaCl) (Nan et al., 2010; Nan et al., 2006). Proteins were loaded onto an NGC Chromatography System (BIO-RAD) and 5 ml HisTrap columns (Cytiva) and eluted with buffer B (20 mM Tris-HCl pH 8.0, 200 mM NaCl, 500 mM immidazole) (Pogue et al., 2018; Nan et al., 2010). Purified proteins were concentrated using Amicon Ultra centrifugal filter units (MilliporeSigma) with a 10 kDa molecular weight cutoff and stored at –80°C.

### LTG activity (RBB) assay

PG was purified following the published method (Zhang et al., 2023; Alvarez et al., 2016; Ramirez Carbo et al., 2024). In brief, *M. xanthus* cells were grown until mid-stationary phase and harvested by centrifugation (6000×*g*, 20 min, 25°C). Supernatant was discarded and the pellet was resuspended and boiled in 1×PBS with 5% SDS for 2 h. SDS was removed by repetitive washing with water and centrifugation (21,000×*g*, 10 min, 25°C). Purified PG from 100 ml culture was suspended in 1 ml 1×PBS and stored at –20°C. RBB labelling of PG was performed essentially as previously described (Uehara et al., 2010; Jorgenson et al., 2014). Purified sacculi were incubated with 20 mM RBB in 0.25 M NaOH overnight at 37°C. Reactions were neutralized by adding equal volumes of 0.25 M HCl and RBB-labeled PG was collected by centrifugation at 21,000×*g* for 15 min. Pellets were washed repeatedly with water until the supernatants became colorless. RBB-labeled sacculi were incubated with purified LtgA or LtgB (1 mg/ml) at 25°C for 12 h. Lysozyme (1 mg/ml) was used as a positive control. Dye release was quantified by the absorption at 595 nm from the supernatants after centrifugation (21,000×*g*, 10 min, 25 °C).

### Immunoblotting

Cells were grown in liquid CYE medium overnight, harvested, and concentrated to OD_600_ 10. 10 μl of cell suspension was mixed with 10 μl of loading buffer and 10 μl of the mixture was subjected to SDS-PAGE. The expression and stability of PAmCherry-labeled proteins were determined by immunoblotting using an anti-mCherry antibody (Rockland Immunochemicals, Inc, Lot 46705) and a goat anti-Rabbit IgG (H+L) secondary antibody, HRP (Thermo Fisher Scientific, catalog # 31460). MreB was detected as the loading control using an anti-MreB serum (Mauriello et al., 2010) and the same secondary antibody. The blots were developed with Pierce ECL Western Blotting Substrate (Thermo Fisher Scientific REF 32109) and a MINI-MED 90 processor (AFP Manufacturing).

## Data Availability

Figure 2—source data 1, Figure 3—source data 1, Figure 4—source data 2, Figure 5—source data 1, Figure 2—figure supplement 2—source data 1, Figure 5—figure supplement 1—source data 2 and Figure 5—figure supplement 1—source data 3 contain the numerical data used to generate the figures. Original data sets are available at Dryad (https://doi.org/10.5061/dryad.q83bk3k0b). The following dataset was generated: BeiyanN 2026Data from: A novel mechanism for morphological change during bacterial sporulation based on programmed peptidoglycan degradationDryad Digital Repository10.5061/dryad.q83bk3k0bPMC1373927142985907

## Additional files

MDAR checklist

Supplementary file 1. Plasmids and primers used in this study.
