# Supplementary material for: A novel mechanism for morphological change during bacterial sporulation based on programmed peptidoglycan degradation
Source: eLife. 2026 Nov 5;14:RP108250. doi: 10.7554/eLife.108250 (PMC13739271; doi:10.7554/eLife.108250)
Supplement: Supplementary file 1 [file elife-108250-fig4-figsupp1-data3.pdf]

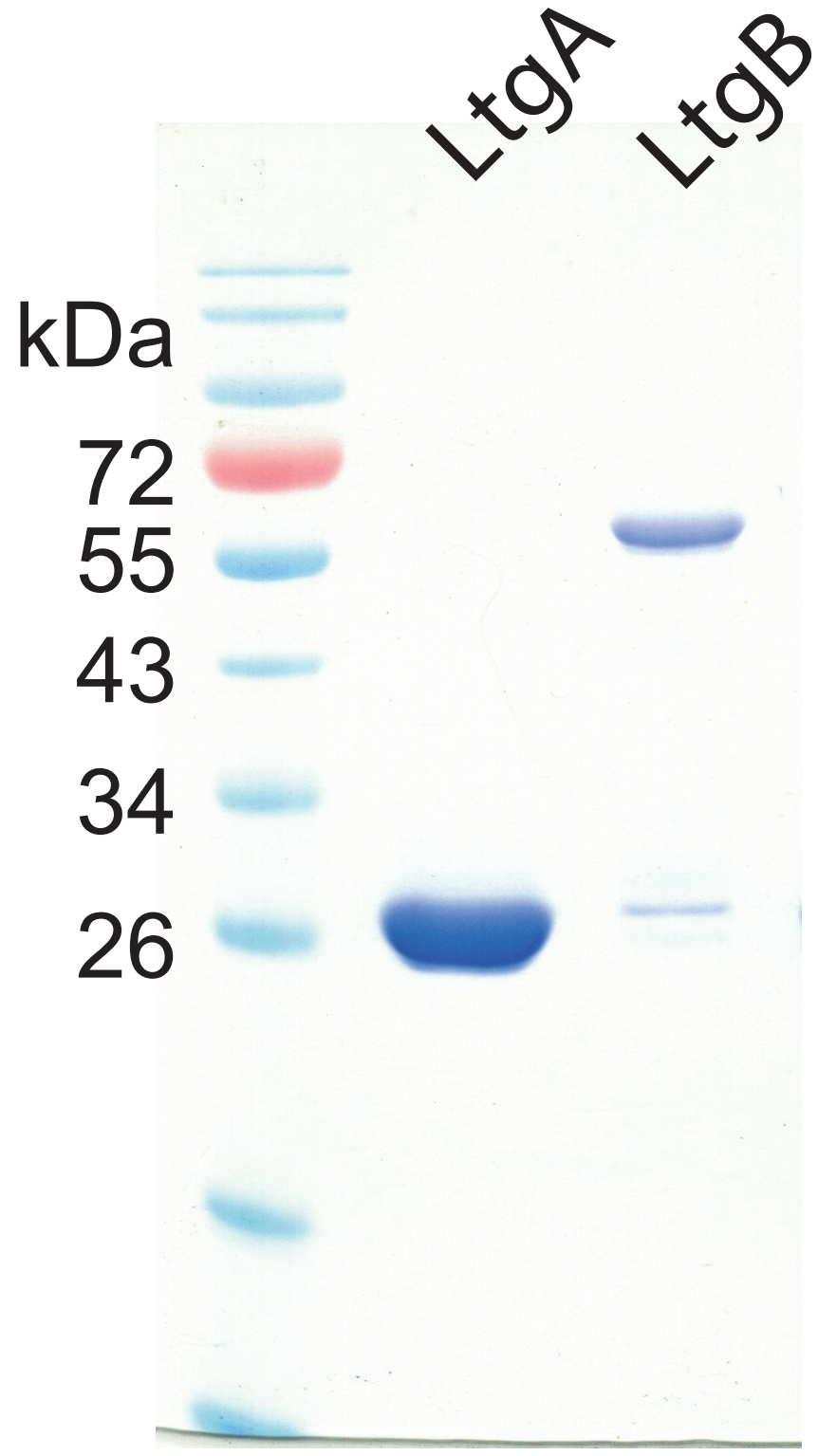
LtgA
LtgB
kDa
72
55
43
34
26
