# Supplementary material for: A novel mechanism for morphological change during bacterial sporulation based on programmed peptidoglycan degradation
Source: eLife. 2026 Nov 5;14:RP108250. doi: 10.7554/eLife.108250 (PMC13739271; doi:10.7554/eLife.108250)
Supplement: Supplementary file 5 [file elife-108250-fig4-figsupp1-data2.zip › Fig_4_source_data_1_labeled/LtgAB_labeled.pdf]

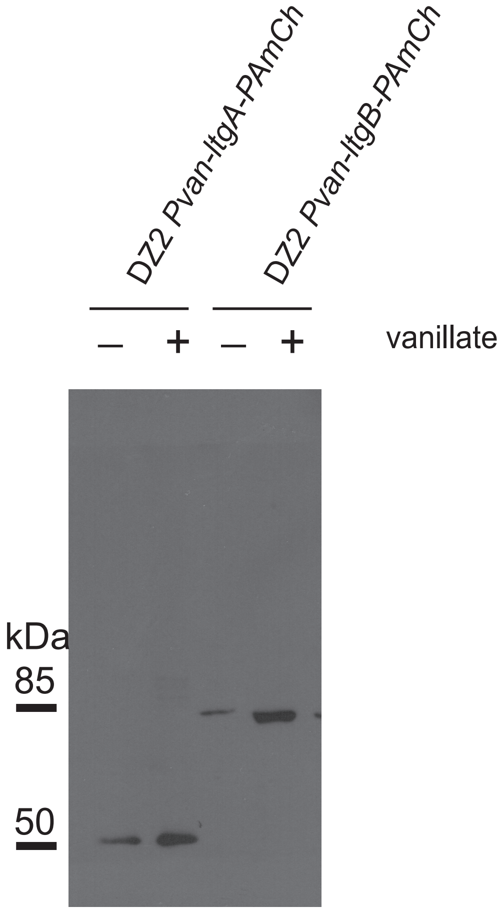
DZ2 Pvan-ltgA-PAmCh
DZ2 Pvan-ltgB-PAmCh
–
+
–
+
vanillate
kDa
85
50
