# Supplementary material for: A novel mechanism for morphological change during bacterial sporulation based on programmed peptidoglycan degradation
Source: eLife. 2026 Nov 5;14:RP108250. doi: 10.7554/eLife.108250 (PMC13739271; doi:10.7554/eLife.108250)
Supplement: Supplementary file 6 [file elife-108250-supp1.docx]

Supplementary File 1

| **Plasmid name** | **Description** | **Source** |
| --- | --- | --- |
| pBJ113 | Plasmid for *M. xanthus* chromosome manipulation through homologous recombination | (Julien *et al.*, 2000) |
| pBJ113-*ΔltgA* | Plasmid for deleting *ltgA* from *M. xanthus* chromosome through homologous recombination | This study |
| pBJ113-*ΔltgB* | Plasmid for deleting *ltgB* from *M. xanthus* chromosome through homologous recombination | This study |
| pBJ113-PAmCherry | Plasmid for labeling *M. xanthus* proteins with PAmCherry through homologous recombination | (Nan *et al.*, 2013) |
| pBJ113-*ltgA-PAmCherry* | Plasmid for labeling LtgA proteins with PAmCherry through homologous recombination | This study |
| pBJ113-*ltgB-PAmCherry* | Plasmid for labeling LtgB proteins with PAmCherry through homologous recombination | This study |
| pMR3629 | Plasmid for overexpressing genes as merodiploids using a vanilate-induced promoter, tet^R^ | (Iniesta *et al.*, 2012) |
| pMR-3629-PAmCherry | Plasmid for overexpressing *PAmCherry*-labeled proteins as merodiploids using a vanilate-induced promoter, tet^R^ | (Zhang *et al.*, 2023) |
| pMR3629-*ltgA* | P*_van_*-*ltgA* in pMR3629, tet^R^ | This study |
| pMR3629-*ltgB* | P*_van_*-*ltgB* in pMR3629, tet^R^ | This study |
| pMR3629-*ltgA-PAmCh* | P*_van_*-*ltgA-PAmCherry* in pMR3629, tet^R^ | This study |
| pMR3629-*ltgB-PAmCh* | P*_van_*-*ltgB-PAmCherry* in pMR3629, tet^R^ | This study |
| pMR3629-*murA* | P*_van_*-*murA* in pMR3629, tet^R^ | This study |
| pET28a-*ltgA* | Plasmid for overexpression of His-tagged LtgA, kan^R^ | This study |
| pET28a-*ltgB* | Plasmid for overexpression of His-tagged LtgB, kan^R^ | This study |

**Plasmids and primers used in this study**

| **Primer name** | **Sequence** | **Usage** |
| --- | --- | --- |
| ltgALF  ltgALR  ltgARF  ltgARR | CCCAAGCTTtgcgcgccacgtgcgcca  CCggatcccacccctcctggcgccg  GGGGTACCgcgctgagcaacaagccatg  GGAATTCggagagggcgcgggcaggctg | Construct pBJ113-*ΔltgA* |
| ltgBLF  ltgBLR  ltgBRF  ltgBRR | CCCAAGcttcatgcaggtgaacgactgcc  CGGATCCgtggtcttcctttcacgacgcg  GGGGTACCgctccgactggggcgccagg  GGAattctctccaggctactccttgcg | Construct pBJ113-*ΔltgB* |
| PAmCHltgALF  PAmCHltgALR  PAmCHltgARF  PAmCHltgARR | CCCAAGCTTTTTCCGGTGCAGGTGCCGGAT  CGGGATCCGCTCTTCTGGCCATTCGTCTTCT  GGGGTACCgcgctgagcaacaagccatg  GGAATTCggagagggcgcgggcaggctg | Construct pBJ113-*ltgA-PAmCherry* |
| PAmCHltgBLF  PAmCHltgBLR  PAmCHltgBRF  PAmCHltgBRR | CCCAAGCTTctatcctcgcgccttcgccac  CGGGATCCgaagctgacaccttccttcgac  GGGGTACCgctccgactggggcgccagg  GGAattctctccaggctactccttgcg | Construct pBJ113-*ltgB-PAmCherry* |
| vanltgAF  vanltgAR | GGGTTTCATATGaggggttggacggtggcgg  GGGGTACCtcagctcttctggccattcgtctt | Construct pMR3629-*ltgA* |
| vanPACltgAF  vanPACltgAR | GGGTTTCATATGaggggttggacggtggcgg  GGGGTACCgctcttctggccattcgtcttc | pMR3629-*ltgA-PAmCh* |
| vanltgBF  vanltgBR | GGGTTTCATATGAGTTGGAGTGGGTGGGTTG  ggggtaccCTAGAAGCTGACACCTTCCTTC | Construct pMR3629-*ltgB* |
| vanPACltgBF  vanPACltgBR | GGGTTTCATATGAGTTGGAGTGGGTGGGTTG  ggggtaccGAAGCTGACACCTTCCTTC | pMR3629-*ltgB-PAmCh* |
| vanmurAF  vanmurAR | GGGTTTCATATGgacaagatcgtcatcaag  GGAATTctacgccttctcgcggcggatgt | Construct pMR3629-*murA* |
| hisltgAF  hisltgAR | CGGGATCCgtgcaggtgccggatggcgcc  cccaagcttcagctcttctggccattcgtct | Construct pET28a-*ltgA* |
| hisltgBF  hisltgBR | CGGGATCCACGGCGCTGGAGGCCGTCCGC  CCCAAGCTTCTAGAAGCTGACACCTTCCTTC | Construct pET28a-*ltgB* |

Iniesta, A.A., Garcia-Heras, F., Abellon-Ruiz, J., Gallego-Garcia, A., and Elias-Arnanz, M. (2012) Two systems for conditional gene expression in *Myxococcus xanthus* inducible by isopropyl-beta-D-thiogalactopyranoside or vanillate. *J Bacteriol* **194**: 5875-5885.

Julien, B., Kaiser, A.D., and Garza, A. (2000) Spatial control of cell differentiation in *Myxococcus xanthus*. *Proceedings of the National Academy of Sciences of the United States of America* **97**: 9098-9103.

Nan, B., Bandaria, J.N., Moghtaderi, A., Sun, I.H., Yildiz, A., and Zusman, D.R. (2013) Flagella stator homologs function as motors for myxobacterial gliding motility by moving in helical trajectories. *Proc Natl Acad Sci U S A* **110**: E1508-1513.

Zhang, H., Venkatesan, S., Ng, E., and Nan, B. (2023) Coordinated peptidoglycan synthases and hydrolases stabilize the bacterial cell wall. *Nature communications* **14**: 5357.
