# Supplementary material for: A novel mechanism for morphological change during bacterial sporulation based on programmed peptidoglycan degradation
Source: eLife. 2026 Nov 5;14:RP108250. doi: 10.7554/eLife.108250 (PMC13739271; doi:10.7554/eLife.108250)
Supplement: Peer review history

# Editor report: eLife Assessment

**DOI:** 10.7554/eLife.108250.4.sa0

**Authors:** Bavesh D Kana

**Affiliations:** https://ror.org/03rp50x72 University of the Witwatersrand South Africa

**Keywords:** Important, Solid

This **important** study reveals the roles of two lytic transglycosylases in the progression of spore formation in the research model species *Myxococcus xanthus*. **Solid** evidence is provided for the roles of these two enzymes in spore formation and for interplay between their functions and peptidoglycan synthetic systems. These findings may have broader implications for studies of peptidoglycan metabolism across a range of species.

# Referee report: Reviewer #2 (Public review)

**DOI:** 10.7554/eLife.108250.4.sa1

The authors initial goal was to demonstrate loss of PG during the slow sporulation process of Myxococcus xanthus, with examination of the PG degradation products in order to implicate possible enzymes involved. Upon finding a predominance of LTG products, they examined sporulation in strains lacking each of the 14 candidate LTGs encoded in the genome, leading to the identification of two sporulation-linked LTGs. An extensive characterization of the roles played by these LTGs. One LTG is responsible for the slow sporulation PG degradation, while another is required for the rapid sporulation process. Interestingly, the "slow" LTG seems to provide an important regulatory brake on the rapid enzyme. Single molecule fluorescent tracking of these enzymes was used to develop a model for their interaction with PG that mimics their observed activity. The rate of PG synthesis activity was also shown to impact the rate of PG degradation, suggesting potential interplay between the synthetic and degradative enzymes.

Strengths:

The genetic analysis to identify sporulation-linked LTGs and their effects on growth sporulation, and spore properties was well done and productive. The fluorescence microscopy to track LTG mobility, presumably tied to activity, produced a convincing argument about the mechanism of regulation of one LTG by another. The authors have responded well to all points of the previous reviews.

# Author comment: Author response

**DOI:** 10.7554/eLife.108250.4.sa2

**Authors:** Carlos Ramirez Carbo, Oihane Irazoki, Srutha Venkatesan, Lauren JS Chen, Haylie A Morales, Assariel J Garcia Avila, Hoi-Ling Cheung, Felipe Cava, Beiyan Nan

**Affiliations:** Texas A&M University College Station United States; https://ror.org/05kb8h459 Umeå University Umea Sweden; Texas A&M University College Station United States; Texas A&M University College Station United States; Texas A&M University College Station United States; Texas A&M University College Station United States; Texas A&M University College Station United States; Texas A&M University College Station United States; Texas A&M University College Station United States

The following is the authors’ response to the previous reviews.

**Public Reviews:**

**Reviewer #2 (Public review):**

The authors initial goal was to demonstrate loss of PG during the slow sporulation process of Myxococcus xanthus, with examination of the PG degradation products in order to implicate possible enzymes involved. Upon finding a predominance of LTG products, they examined sporulation in strains lacking each of the 14 candidate LTGs encoded in the genome, leading to the identification of two sporulation-linked LTGs. An extensive characterization of the roles played by these LTGs. One LTG is responsible for the slow sporulation PG degradation, while another is required for the rapid sporulation process. Interestingly, the "slow" LTG seems to provide an important regulatory brake on the rapid enzyme. Single molecule fluorescent tracking of these enzymes was used to develop a model for their interaction with PG that mimics their observed activity. The rate of PG synthesis activity was also shown to impact the rate of PG degradation, suggesting potential interplay between the synthetic and degradative enzymes.

Strengths:

The genetic analysis to identify sporulation-linked LTGs and their effects on growth sporulation, and spore properties was well done and productive. The fluorescence microscopy to track LTG mobility, presumably tied to activity, produced a convincing argument about the mechanism of regulation of one LTG by another. The authors have responded well to most points of the previous review.

Weaknesses:

While the impact of LTGs on sporulation was clearly demonstrated, the PG analysis that resulted in the study of LTGs raised some important unanswered questions. The analyses suggest that the PG is degraded to quite small fragments, which would normally be lost during the purification of PG. The conclusions concerning the PG degradation during sporulation needs to be clarified, as described below. The authors suggest a "new mechanism of sporulation" when they have actually simply identified an important factor (PG degradation by LTGs) within a complex "process of sporulation". This needs to be reflected also in title of the paper.

We have addressed the reviewer’s concerns and updated the text.

**Recommendations for the authors:**

**Reviewer #2 (Recommendations for the authors):**

(1) Lines 100-125: I am still concerned about clarity in the description of the muropeptides from spores. The claim is that 90% of the recovered muropeptides are anhydro LTG products. If LTGs had truly cleaved so many of the glycosidic bonds such that 90% of the muramic acid was now in the anhydro form, then all of the PG in the spores would be in small fragments (dimers and trimers containing 4-6 sugars and 8-12 amino acids), and these would be soluble and lost during purification. Whatever the form of the PG, it must not be easily soluble, so it must be either larger than that or bound to something else. The fact that the muropeptides are solubilized by muramidase indicates that there are some NAM-NAG bonds remaining, and cleavage of these should release some non-anhydro muropeptides. The spore muropeptide chromatograms have a large, late "mound" of UV-absorbing material that was released by muramidase digestion, and two of the identified anhydro products are present in this mound. It is not clear how these two anhydro products were quantified within this mound and what other (presumably) muropeptide species might be present in this mound. Some explanation of how these two muropeptides were quantified is needed.

We thank the reviewer for this careful point. As the reviewer notes, our purification procedure recovers only sedimentable PG, and any PG fragments solubilized by LTG activity would be lost during the washing steps and therefore not represented in our analysis. We have now explicitly acknowledged this limitation and discussed its implications in the revised text:

“Consequently, any PG fragments solubilized by LTG activity during sporulation would be lost at this stage, and the muropeptides we detect derive from the more highly crosslinked material that survives the procedure.

“Despite the overall decline in identified muropeptides, anhydro-muropeptides a minor component of vegetative PG were enriched in both spore types (Figure 1).”

I feel that the authors need to address some of this uncertainty in the results and discussion. They might be able to say that anhydro-muropeptides represent 90% of the "identified muropeptides" but need to acknowledge that there is a great deal of unidentified material released by the muramidase digestion. The data might also indicate that the LTG activity solubilizes much of the PG, which is lost, and results in recovery of only highly cross-linked muropeptides that might survive the PG purification process.

We agree that two of the identified anhydro species elute within a broad, unresolved region of the chromatogram that accounts for a substantial fraction of the muramidase-released material. Because reliable assignment and quantification of individual components within this region is not possible, we concluded that expressing anhydro-muropeptides as a fraction of the total identified muropeptides could be misleading. We have therefore removed the quantitative statement and now describe the enrichment of anhydromuropeptides in spores relative to vegetative cells qualitatively:

“The identified muropeptides, however, represent only a part of the material released by muramidase from spores: a substantial, as a late-eluting portion of the chromatogram could not be assigned, and its composition remains unknown.”

This would not eliminate the conclusion that "their abundance in spores indicates that certain LTGs must play essential roles (perhaps change to "might play important roles") in M. xanthus sporulation", which leads to the remaining studies in the paper.

Following the reviewer's suggestion, we have softened the conclusion of this section to state that LTGs "may play important roles" in sporulation.

(2) The authors have changed the statement about a "new mechanism of sporulation" at the beginning of the discussion, but this language is still in the paper title. Something more like "Programmed peptidoglycan degradation plays an important role in Myxococccus sporulation"

Following the reviewer's suggestion, we changed the title to “A novel mechanism for morphological change during bacterial sporulation based on programmed peptidoglycan degradation”.
